# Combination therapy with oncolytic virus and T cells or mRNA vaccine amplifies antitumor effects

**DOI:** 10.1038/s41392-024-01824-1

**Published:** 2024-05-03

**Authors:** Rao Fu, Ruoyao Qi, Hualong Xiong, Xing Lei, Yao Jiang, Jinhang He, Feng Chen, Liang Zhang, Dekui Qiu, Yiyi Chen, Meifeng Nie, Xueran Guo, Yuhe Zhu, Jinlei Zhang, Mingxi Yue, Jiali Cao, Guosong Wang, Yuqiong Que, Mujing Fang, Yingbin Wang, Yixin Chen, Tong Cheng, Shengxiang Ge, Jun Zhang, Quan Yuan, Tianying Zhang, Ningshao Xia

**Affiliations:** 1 State Key Laboratory of Vaccines for Infectious Diseases, Xiang An Biomedicine Laboratory, Department of Laboratory Medicine, School of Public Health & School of Life Sciences, Xiamen University, Xiamen, 361102 Fujian China; 2 National Institute of Diagnostics and Vaccine Development in Infectious Diseases, Xiamen, 361102 Fujian China

**Keywords:** Cancer therapy, Drug development

## Abstract

Antitumor therapies based on adoptively transferred T cells or oncolytic viruses have made significant progress in recent years, but the limited efficiency of their infiltration into solid tumors makes it difficult to achieve desired antitumor effects when used alone. In this study, an oncolytic virus (rVSV-LCMVG) that is not prone to induce virus-neutralizing antibodies was designed and combined with adoptively transferred T cells. By transforming the immunosuppressive tumor microenvironment into an immunosensitive one, in B16 tumor-bearing mice, combination therapy showed superior antitumor effects than monotherapy. This occurred whether the OV was administered intratumorally or intravenously. Combination therapy significantly increased cytokine and chemokine levels within tumors and recruited CD8^+^ T cells to the TME to trigger antitumor immune responses. Pretreatment with adoptively transferred T cells and subsequent oncolytic virotherapy sensitizes refractory tumors by boosting T-cell recruitment, down-regulating the expression of PD-1, and restoring effector T-cell function. To offer a combination therapy with greater translational value, mRNA vaccines were introduced to induce tumor-specific T cells instead of adoptively transferred T cells. The combination of OVs and mRNA vaccine also displays a significant reduction in tumor burden and prolonged survival. This study proposed a rational combination therapy of OVs with adoptive T-cell transfer or mRNA vaccines encoding tumor-associated antigens, in terms of synergistic efficacy and mechanism.

## Introduction

With the growing comprehension of immune activity within tumor sites, immunotherapy has garnered significant attention as a potent strategy for cancer treatment, resulting in a significant shift in both cancer research and clinical trials. The primary objective of tumor immunotherapy is to stimulate the host’s antitumor immunity, establish a immunosensitive microenvironment, and ultimately accomplish tumor shrinkage while enhancing the overall survival rate of patients.^1,2^

The implementation of chimeric antigen receptor (CAR) -T-cell therapy, a form of adoptively transferred T cells therapy, in the treatment of B-cell malignancies has surpassed expectations.^3,4^ However, unlike hematological malignancies, solid tumors pose significant challenges. Adoptively transferred T cells must traverse a long distance to penetrate the dense and resilient matrix and establish interactions with chemokine receptors. Once they reach the tumor microenvironment (TME), most T cells encounter obstacles and immunosuppressive factors that hinder their expansion, infiltration, and ability to induce tumor-specific cytotoxicity.^5–7^ The limited effectiveness of T-cell-based monotherapy in treating solid tumors suggests that additional adjuvant therapies are necessary to overcome these resistance mechanisms and extend the application of adoptively transferred T cells therapy to solid tumors.

On 2nd Oct 2023, the Nobel Prize in Physiology or Medicine was awarded to two developers of mRNA vaccines. The accelerated availability of mRNA coronavirus disease 2019 (COVID-19) vaccines has provided valuable health protection, but it only scratches the surface of the vast potential of mRNA vaccines. Furthermore, mRNA vaccines have a compelling application beyond preventing infectious diseases: cancer vaccines.^8–10^ The potential of mRNA vaccines is being further realized through the optimization of their structure, stability, and delivery methods, alongside advancements in personalized design and preparation processes.^11–13^ Currently, dozens of clinical trials are testing the safety and effectiveness of mRNA vaccines against a variety of cancers, including pancreatic, colorectal, and melanoma. Additionally, certain trials are investigating the synergistic potential of combining mRNA vaccines with immunomodulatory drugs to enhance the body’s immune response towards tumors.^14–16^ A specific cytokine-encoding mRNA vaccine has demonstrated the capacity to significantly diminish tumor volume and prolong the survival of mice, consequently, clinical evaluation of this particular cytokine-encoding mRNA formulation is currently in progress.^17^ In pancreatic cancer patients, the administration of an mRNA vaccine that encodes numerous neoantigens enhances the immune system’s response, during an 18-month follow-up period, approximately half of the patients who received a combination of immunotherapy, chemotherapy, and an mRNA cancer vaccine exhibited a successful immune response to the vaccine and remained free of cancer recurrence.^18^ Clinical and preclinical trials have demonstrated promising outcomes for personalized mRNA-based cancer vaccines, indicating a potential paradigm shift in cancer treatment. It is also crucial to acknowledge that a standalone tumor mRNA vaccine is incapable of fully tackling all the obstacles encountered in immunotherapy. These challenges encompass multiple immune escape mechanisms employed by tumor cells, interference and inhibition resulting from the TME, as well as tumor invasion.

Oncolytic virotherapy is another promising treatment for solid tumors due to its selective nature, optimal immunogenicity, and ability to deliver transgenes directly to the tumor site in a targeted manner.^19^ The anticancer effects of oncolytic viruses (OVs) are primarily achieved through directly lysing tumor cells and counteracting the immunosuppressive microenvironment within the tumor.^20,21^ In addition, OVs can be genetically modified to express specific genes within the tumor environment, thereby enhancing their oncolytic properties and promoting antitumor immune responses.^22^ Despite their immense potential in cancer therapy and their emergence as a novel branch of tumor treatment, the clinical application of OVs is still confronted with several challenges, as highlighted in previous studies.^23,24^ In current clinical trials, intratumoral injection is considered the most effective and safe route of administration for OVs, particularly for surface or localized tumors. However, systemic administration, such as intravenous injection, holds greater clinical application prospects and commercial value, especially for the treatment of metastatic tumors.^25,26^ Although OVs have shown limited success as standalone therapies, they have the potential to act synergistically with other immunotherapies, such as adoptive cellular therapy.^27^ There are several mechanisms by which OVs can enhance the efficacy of immunotherapy.^21,28,29^ Firstly, OVs can reverse tumor immunosuppression by releasing danger signals, promoting the trafficking, proliferation, and persistence of T cells within the TME. Secondly, OVs have the ability to lyse tumor cells, releasing relevant antigens. This helps to counteract tumor escape mechanisms caused by antigen loss. Additionally, OVs can serve as carriers to deliver chemokines or cytokines, further augmenting the antitumor function of T cells. Given these observations, we hypothesized that OVs could amplify the antitumor effects of adoptively transferred T cells or tumor mRNA vaccine and sought to define the mechanisms underlying this synergistic effect.

## Results

### OV delivery of GP33 to solid tumor cells redirect the activity and cytotoxicity of P14 T cells in vitro

**Fig. 1:**
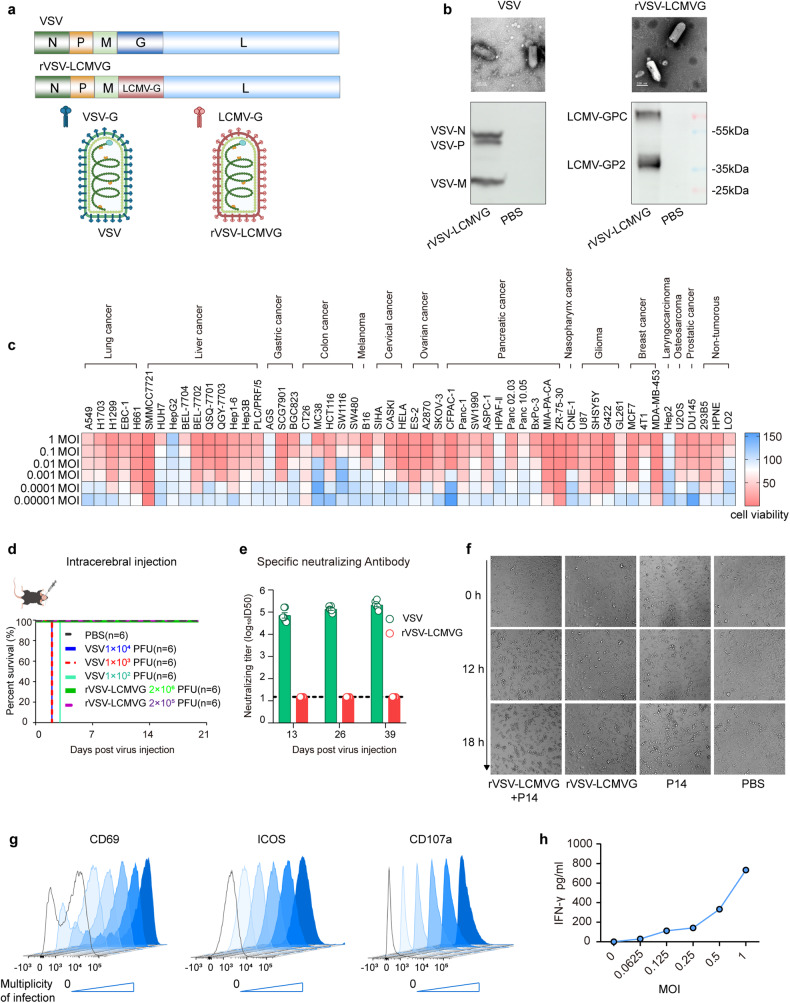
Characterization of rVSV-LCMVG, which could effectively deliver GP33 to tumor cells to direct the activation and cytotoxicity of P14-TCR-T cells in vitro. **a** Schematic of oncolytic virus rVSV-LCMVG showing the G gene of VSV genome replaced by the G gene of LCMV. **b** Electron micrographs of VSV and rVSV-LCMVG, and identification of N, P, M, LCMVG protein expression via western blot analysis. **c** Murine and human cancer cells were infected with rVSV-LCMVG at the indicated MOIs. Cell viability was analyzed at 48 h after virus infection, using CCK8 cell viability assay kits. **d** Inoculation with wild-type virus VSV or rVSV-LCMVG via intracranial injection, to monitor the survival of mice. **e** Inoculation 1 × 10^7^ PFU of rVSV-LCMVG or VSV by intravenous injection, one dose every three days and every three injections is a course of treatment, the blood is collected to detect the content of neutralizing antibodies in serum. **f** Results of B16-OVA tumor cell killing assay, as visualized by phase-contrast microscopy. Representative images are shown. Scale bar, 50 μm. **g** Expression of cell surface CD69, ICOS, and CD107a on P14 cells after 16-hour coculture with B16-OVA tumor cells in the presence or absence of the indicated MOI of rVSV-LCMVG. **h** IFN-γ production in supernatants measured by enzyme-linked immunosorbent assay (ELISA) collected from cocultures with P14 at indicated MOIs for 16 h

The vesicular stomatitis virus (VSV) is a potential oncolytic viral vector.^30^ In order to reduce neurotoxicity while retaining the lytic potency and wide-ranging tumor tropism of VSV,^31–33^ the G protein of VSV was replaced with the G protein of Lymphocytic Choriomeningitis Virus (LCMV), and the modified recombinant virus was named rVSV-LCMVG (Fig. 1a). Electron microscopy showed that rVSV-LCMVG maintained the original bullet-shaped particles, and the expression of viral proteins VSV-N, VSV-P, and VSV-M was detectable in anti-VSV-Rat serum, and the presence of LCMVG protein could be detected using anti-LCMVG monoclonal antibodies (mAb) (Fig. 1b). rVSV-LCMVG displayed remarkable cytotoxic effects on diverse tumor cell lines, even at multiplicity of infection (MOI) levels below 0.01. Notably, it exhibited particularly strong killing effects on liver and lung cancer cell lines, highlighting its potential as an oncolytic virus (Fig. 1c and Supplementary Fig. 1a). IC50 of rVSV-LCMVG for various cancer cell lines was in Supplementary Fig. 1b. To evaluate its efficiency in infecting and producing LCMVG in tumor cells, we infected B16-OVA cells with rVSV-LCMVG at different multiplicities of infection (MOI), 16 h later the expression of VSV-P and LCMVG could be detected (Supplementary Fig. 1c, d). We also conducted an assessment of the proportion of tumor cells that exhibited positivity towards LCMVG and VSV-P antigens following exposure to different rVSV-LCMVG MOIs for 12, 24, and 48 h. Flow cytometry analysis demonstrated a significant increase in the proportion of tumor cells expressing LCMVG and VSV-P, which was dependent on the MOI (Supplementary Fig. 1e). Compared to the wild-type VSV, the rVSV-LCMVG also exhibited significantly enhanced safety. The intracranial injection of 1 × 10^2^ plaque-forming units (PFU) of the wild-type VSV led to the mortality of all mice, whereas all mice that received 1 × 10^6^ PFU of rVSV-LCMVG survived (Fig. 1d and Supplementary Fig. 1f). Compared to the wild-type VSV, the modified rVSV-LCMVG demonstrated a low propensity to induce the production of neutralizing antibodies after multiple intravenous doses (Fig. 1e). C57BL/6 J mice were intravenously injected with rVSV-LCMVG at 1 × 10^7^ PFU and no significant weight loss between day 1 to day 30 postinjection when compared with PBS-treated controls (Supplementary Fig. 2a, b). To provide a more detailed analysis of potential toxicity, the same doses of rVSV-LCMVG were injected intravenously and serum ALT (Alanine aminotransferase) well as AST (Aspartate aminotransferase) were determined. Both levels were not elevated in any of the group throughout the observation period, indicating a lack of potential toxicity (Supplementary Fig. 2c, d). Quantitative RT-PCR showed that the rVSV-LCMVG virus genome decreased gradually with the extension of infection time in the blood, heart, liver, spleen, lung, kidney and brain of the treated animals (Supplementary Fig. 2e). These results suggested that rVSV-LCMVG exhibited safety as an oncolytic virus in the treatment of tumors, and it could be employed in a multi-injection, multi-course administration strategy to mitigate the influence of neutralizing antibodies.

To assess the susceptibility of rVSV-LCMVG-infected cells to specific T-cell-mediated killing, B16-OVA cells were infected with rVSV-LCMVG for 16 h and co-cultured with P14 cells, which can recognize LCMV-GP33, at effector: target (E:T) ratios of 1:1. The group that underwent combined rVSV-LCMVG infection and P14 cells coculture exhibited significantly higher levels of killing compared to B16-OVA cells infected with rVSV-LCMVG alone or co-cultured with P14 cells alone at each time point (Fig. 1f). CD69 and ICOS were employed as T-cell activation surface markers, while CD107a levels on the cell surface and the concentration of interferon-γ (IFN-γ) in the supernatant were used to assess P14 cells function. The activity of P14 T cells, which were co-cultured for 16 h with rVSV-LCMVG infected B16-OVA cells, exhibited robustness that was dependent on the rVSV-LCMVG MOI (Fig. 1g, h). These findings suggest that OVs have the capability to deliver antigens, in this case LCMVG to tumors and enhance antigen-specific T-cell-mediated antitumor responses.

### Tumor-reactive and OV-reactive P14 T cells conferred stronger antitumor immunity

**Fig. 2:**
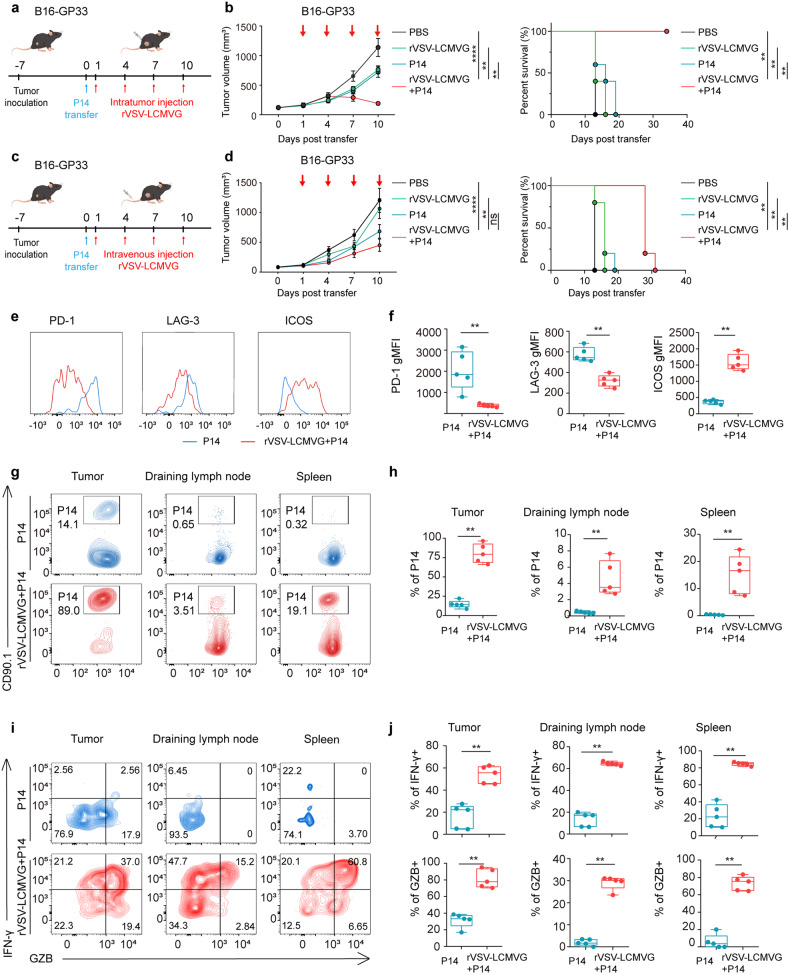
Antitumor efficacy of rVSV-LCMVG combined with P14 cells in B16-GP33 tumor models. **a** Schematic of B16-GP33 tumor-bearing mice treated with rVSV-LCMVG and P14 T cells. **b** Tumor volumes are shown as mean values with SEM (*n* = 5 per group). Survival curves of C57BL/6 J mice from the experiment described in **a** are shown. *p < 0.05; **p < 0.01; ***p < 0.001; ****p < 0.0001, based on two-way ANOVA with post hoc Holm–Sidak test; survival analysis was conducted using log rank test. **c** Schematic of the treatment was the same as that in (**a**), except oncolytic virus was administered intravenously. **d** Tumor volumes are shown as mean values with SEM (*n* = 5 per group). Survival curves of C57BL/6 J mice from the experiment described in **c** are shown. **e** Representative flow cytometri**c** analysis showing abundance of inhibitory receptors (PD-1 and LAG3) and activation molecules ICOS on tumor-infiltrating P14 cells isolated from the tumor. **f** Quantification (geometric mean of fluorescence intensity) of the expression levels of PD-1, LAG3 and ICOS on tumor-infiltrating P14 cells. Each dot represents one mouse. **g** Flow cytometry plot showing the fraction of P14 (CD90.1^+^) cells in the total CD8^+^ T-cell gate from the tumor, draining lymph node, and spleen of a representative mouse. **h** Quantification of P14 in **g**. Each dot represents one mouse. **i** Representative intracellular staining for the cytokines IFN-γ and GZMB. **j** Summary of cytokine production by P14 cells. Each dot represents one mouse. Horizontal bars show the minimum and maximum values (*p < 0.05, **p < 0.01, ***p < 0.001, ****p < 0.0001, ns means not significant based on the Mann Whitney test)

Given the limitations of OVs and adoptively transferred T-cell monotherapy for the treatment of solid tumors, we conducted a study to investigate the potential of combination therapy. In this study, we utilized the B16-GP33 melanoma model, which expresses the exogenous antigen GP33, to assess the effectiveness of combination therapy involving rVSV-LCMVG and P14 cells. Once the tumor size reached approximately 100 mm^3^ following the subcutaneous injection of B16-GP33 cells, we transferred P14 cells (2 × 10^6^ cells per mouse) on day 0, relative to treatment. The next day, on day 1, the tumors were intratumorally (i.t.) injected with rVSV-LCMVG 1 × 10^7^ PFU per dose for every 3 days for 12 consecutive days (Fig. 2a). To investigate the impact of each component of combinatorial treatment on B16-GP33 tumor growth, groups of mice with established tumors were assigned to four treatment groups: PBS (control), rVSV-LCMVG alone, P14 alone, or combination therapy with rVSV-LCMVG and P14. Tumor growth was assessed every three days. As expected, mice treated with either P14 cells alone or rVSV-LCMVG alone exhibited slower tumor growth compared to the control group treated with PBS. Combination therapy resulted in significant tumor regression and a substantial increase in survival time. 10 days after the injection of rVSV-LCMVG, mice treated with either P14 T cells or rVSV-LCMVG alone showed a moderate reduction in tumor volume, whereas the P14 combined with rVSV-LCMVG group completely eliminated the tumor after 19 days. Furthermore those receiving dual treatment survived for more than 35 days until the conclusion of the experiment (Fig. 2b and Supplementary Fig. 3a). Therefore, in an attempt to address the limited therapeutic impact of systemically administering OVs, we sought to enhance the therapeutic efficacy by combining rVSV-LCMVG with P14 through intravenous injection at an equivalent dosage to the previous intratumoral injection. We transferred P14 into B16-GP33-bearing mice one day before the administration of rVSV-LCMVG (Fig. 2c). Tumors that progressed in the group receiving intravenous administration of rVSV-LCMVG maintained similar levels compared with those in the group receiving PBS treatment. However, when P14 was combined with intravenous administration of rVSV-LCMVG, there was a significant improvement in tumor treatment efficacy and survival rates. (Fig. 2d and Supplementary Fig. 3b).

To investigate the mechanisms by which rVSV-LCMVG enhances the effects of adoptive T-cell therapy, we examined the number, phenotype, and function of P14 cells in tumors and the peripheral region using flow cytometry. This analysis was performed after intratumoral administration of two doses of the oncolytic virus on the fifth day. We observed changes in the functions of P14 cells in the P14 transferred group. These cells upregulated inhibitory receptors, such as PD-1 and LAG3, downregulated the expression of the co-stimulatory molecule ICOS. Furthermore, P14 cells in the P14 combined with rVSV-LCMVG group showed reduced expression levels of LAG3 and PD-1, as well as increased expression levels of ICOS (Fig. 2e, f). Additionally, P14 cells obtained from the tumors, including draining lymph nodes and spleen, showed higher abundance in the group treated with a combination of P14 cells and rVSV-LCMVG, compared to the group treated with P14 cells alone. (Fig. 2g, h). P14 cells in the P14 transferred group also exhibited lower levels of IFN-γ and GZMB upon ex vivo restimulation. P14 cells in the P14 combined with rVSV-LCMVG group produced more IFN-γ and GZMB upon restimulation ex vivo, compared with P14 transferred group (Fig. 2i, j), indicating improved P14 cells function in the TME. Consistent with the rapid development of P14 cells dysfunction, the aggressive growth of the B16-GP33 melanoma tumor could only be controlled by the adoptive transfer of P14 cells during the early stages of the disease. We also analyzed the expression of 36 soluble cytokines and chemokines in the B16-GP33 tumor using the Luminex beads method, in addition to detecting specific T cells cytokine production. In tumors treated with the combination therapy of rVSV-LCMVG and P14 cells, the intratumor levels of IFN-γ, TNF-α, IL-2, IL-12, IL-15, and GM-CSF were significantly higher compared to the single-strategy group. These elevated levels of cytokines could induce tumor regression and stimulate systemic immunity. Furthermore, treatment with the combination therapy also led to significantly higher levels of CCL5 and CXCL10, which may attract inflammatory cells to the injection site (Supplementary Fig. 3c). Therefore, the continuous injections of rVSV-LCMVG after infiltration of P14 cells into the tumor altered the cytokine profile in the TME, as the infiltrating cells responded to rVSV-LCMVG treatment. Correspondingly, the combination of P14 and rVSV-LCMVG treatment significantly enhanced the survival rates of mice bearing B16-GP33 tumors. These findings suggest that the improved tumor control observed after the combination therapy was mediated by the oncolytic virus, which promotes greater infiltration of T cells and enhances their antitumor capacity within the reconstituted tumor immune microenvironment. Consequently, the combination therapy with oncolytic virus has a profound impact on the responses to adoptively transferred T-cell therapy.

### OVs enhanced the antitumor function of tumor-specific T cells and OV-specific T cells

**Fig. 3:**
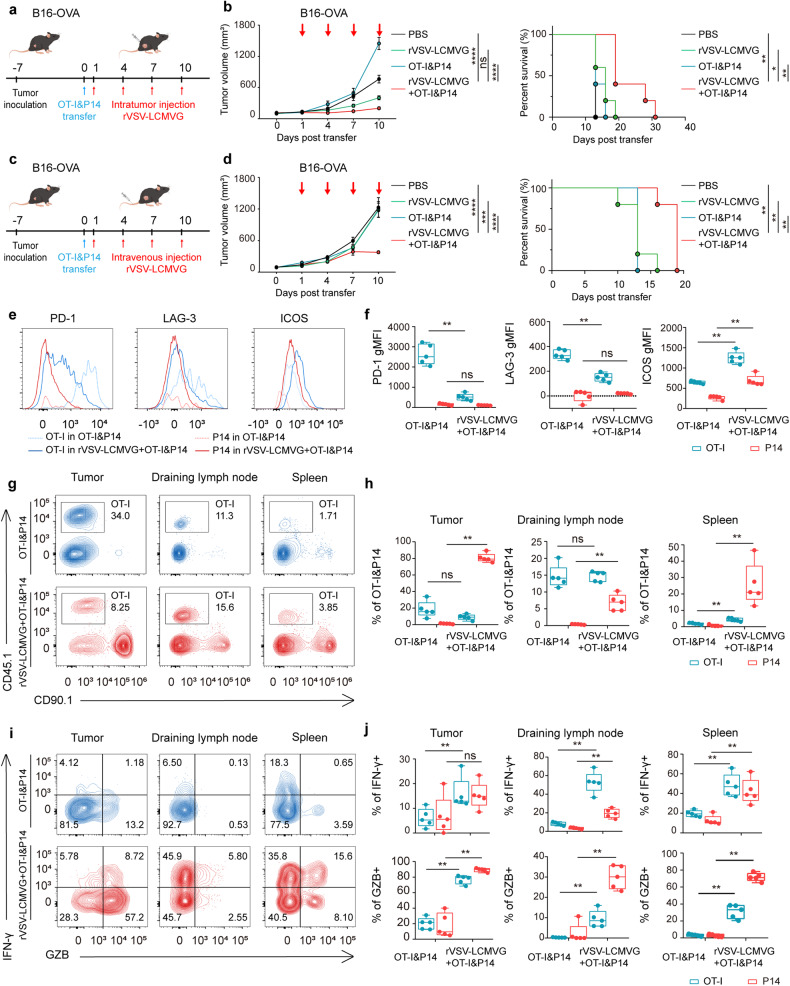
Antitumor efficacy of combination therapy of rVSV-LCMVG and P14, OT-I cells in B16-OVA tumor models. **a** Schematic of B16-OVA tumor-bearing mice treated with rVSV-LCMVG and OT-I and P14 T cells. **b** Tumor volumes are shown as mean values with SEM (*n* = 5 per group). Survival curves of C57BL/6 J mice in **a** are shown. *p < 0.05; **p < 0.01; ***p < 0.001; ****p < 0.0001, based on two-way ANOVA with post hoc Holm–Sidak test; survival analysis was conducted by log rank test. **c** Schematic of the treatment was the same with **a**, except oncolytic virus was administered intravenously. **d** Tumor volumes are shown as mean values with SEM (*n* = 5 per group). Survival curves of C57BL/6 J mice from the experiment are described in **c**. **e** Representative flow cytometric analysis showing abundance of inhibitory receptors (PD-1 and LAG3) and activation molecules ICOS on tumor-infiltrating OT-I and P14 cells isolated from the tumor. **f** Quantification (geometric mean of fluorescence intensity) of the expression levels of PD-1, LAG3, and ICOS in tumor-infiltrating OT-I and P14 cells. Each dot represents one mouse. **g** Flow cytometry plot showing the fraction of OT-I (CD45.1^+^) cells in the total CD8^+^ T-cell gate, in the tumor, draining lymph node, or spleen of a representative mouse. **h** Quantification of the OT-I and P14 in the total CD8^+^ T-cell gate, in the tumor, draining lymph node, or spleen. Each dot represents one mouse. **i** Representative intracellular staining for the cytokines IFN-γ and GZMB. **j** Summary of cytokine production by OT-I and P14 cells upon restimulation with cognate peptides. Each dot represents one mouse. Horizontal bars show the minimum and maximum values (*p < 0.05, **p < 0.01, ***p < 0.001, ****p < 0.0001, ns means not significant based on the Mann Whitney test)

To further investigate the changes of tumor-specific and virus-specific T cells when combined oncolytic virus therapy, the antitumor activity of this combination approach was tested in a syngeneic tumor model using C57BL/6 J mice bearing subcutaneous B16-OVA tumors, a melanoma cell line engineered to express the exogenous antigen chicken ovalbumin (OVA). The melanoma cell line B16-OVA was subcutaneously injected firstly, and when the tumor size reached approximately 100 mm^3^, an appropriate amount of P14 and OT-I (2 × 10^6^ cells per mouse) were transferred on day 0 (relative to treatment). On the next day, followed by four doses of oncolytic virus therapy, one dose every three days, rVSV-LCMVG 1 × 10^7^ PFU/dose (Fig. 3a). A modest decrease in tumor burden and an enhancement in overall survival were observed in mice with intratumoral injection of four doses of 1 × 10^7^ PFU rVSV-LCMVG. Furthermore, significant tumor suppression was observed in the group receiving combined treatment, leading to a more effective extension of the survival rates of mice. (Fig. 3b and Supplementary Fig. 4a). In addition to intratumoral administration, we also assessed the therapeutic efficacy of intravenous administration of the oncolytic virus in conjunction with T cells in the B16-OVA tumor model (Fig. 3c). Compared to the single treatment group, the co-administration of T cells along with intravenous administration of oncolytic virus demonstrated a notable therapeutic effect in inhibiting tumor growth and extending the lifespan of mice (Fig. 3d and Supplementary Fig. 4b). Tumor-specific OT-I T cells isolated from the tumors exhibited high levels of PD-1 and LAG3, whereas bystander P14 cells isolated from the same tumors displayed much lower levels of these markers. Furthermore, the expression of PD-1 and LAG3 decreased in OT-I cells when combined with rVSV-LCMVG treatment, while the expression of ICOS increased (Fig. 3e, f). Furthermore, 5 days after transfer, both OT-I and P14 cells infiltrated the tumors in the OT-I&P14 treatment group, with OT-I cells showing higher levels of infiltration compared to non-specific P14 cells in B16-OVA tumors, while enhanced recruitment of virus-specific P14 T cells was observed in the presence of rVSV-LCMVG. Additionally, tumors, draining lymph nodes, and spleen exhibited a similar trend (Fig. 3g, h and Supplementary Fig. 4c). In addition, OT-I tumor-infiltrating lymphocytes (TILs) demonstrated decreased production of IFN-γ compared to OT-I cells in the spleen. However, when mice were treated with a combination of OT-I and P14 cells along with rVSV-LCMVG, the levels of cytokines secreted by both cells significantly increased in tumors, draining lymph nodes, and spleens (Fig. 3i, j and Supplementary Fig. 4d). The findings demonstrate that the tumor-specific T cells infiltrating the tumor site show signs of exhaustion. Nevertheless, when administered in combination with the oncolytic virus rVSV-LCMVG therapy, the exhaustion phenotype of the tumor antigen-specific T cells (OT-I) can be reversed. Additionally, the detection of cytokines revealed an augmented secretion by the combined OT-I cells and oncolytic virus, thereby intensifying the antitumor effect.

Next, multiplex immunofluorescence imaging was performed to better characterize structures within the tumor and draining lymph nodes at the cell-cell interaction level. When examining the draining lymph nodes, it was observed that the ratio of OT-I and P14 T cells in the combined treatment group was significantly higher compared to the single treatment group (Supplementary Fig. 5a, b). In the tumor region, there was a notable increase in the overall infiltration of CD8 T cells, OT-I, and P14 T cells in tumors treated with T cells in conjunction with rVSV-LCMVG, as opposed to the adoptive transfer of OT-I and P14 alone. Moreover, we found a close colocalization of PD-1 and CD8 expression in tumors, with a relatively low expression level of PD-1 in the combined treatment group (Supplementary Fig. 5c, d). This finding was consistent with previous flow cytometry results.

### Transcriptional signature of OT-I and P14 TILs

**Fig. 4:**
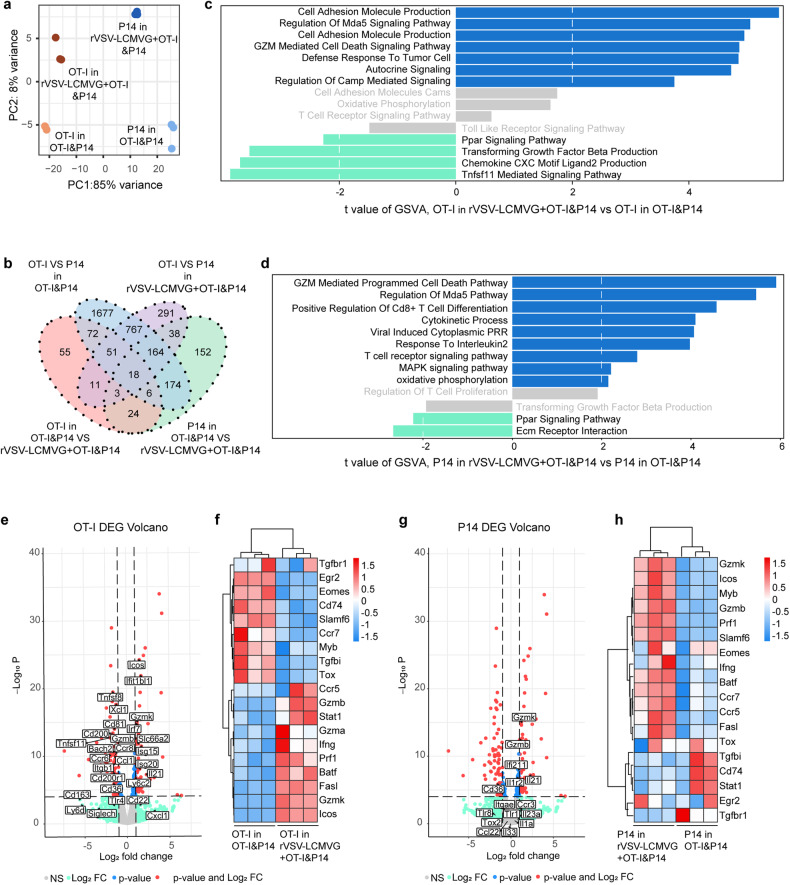
OT-I and P14 T cells have distinct transcriptional profile when combined with rVSV-LCMVG in B16-OVA. Transcriptome kinetics of OT-I and P14 T cells following a transfer of OT-I and P14 T cells into B16-OVA tumor-bearing mice on day 0, then followed by two doses of rVSV-LCMVG intratumorally administered on day 1 and day 4 (or not). For bulk RNA-seq, OT-I and P14 T cells were harvested and sorted on day 5. **a** Principal components analysis of mRNA matrix from all cells in combination treatment group or the monotherapy. **b** Venn-diagram showing differential RNA-seq peaks for OT-I and P14 T cells in combination treatment group compared to the monotherapy. **c** Differences in pathway activity scores of OT-I T cells between the combination treatment and monotherapy groups. **d** Differences in pathway activity scores of P14 T cells between the combination treatment and monotherapy groups. **e** Volcano plot of differentially expressed genes fold changes in OT-I T cells between the combination treatment and monotherapy groups. **f** Heatmap depicting representative protein export genes of OT-I T cells from the combination treatment and monotherapy groups. **g** Volcano plot of differentially expressed genes fold changes in P14 T cells between the combination treatment and monotherapy groups. **h** Heatmap depicting representative protein export genes of P14 T cells from the combination treatment and monotherapy groups

The aforementioned studies demonstrated distinct proliferative and differentiation responses of tumor-specific T-cell OT-I and virus-specific T-cell P14 to various treatments. Thus, it became crucial to explore the disparities in the transcriptional signatures of these T cells expanded after adoptive transfer of T-cell monotherapy or combined oncolytic virus therapy. To accomplish this, RNA sequencing (RNA-seq) analysis was conducted on the sorted tumor-specific and virus-specific T cells obtained from mice in both treatment groups with B16-OVA tumors. The RNA-seq results indicated significant alterations in the gene expression profiles of both tumor-specific and virus-specific T cells in mice treated with combination therapy as opposed to those treated with monotherapies (Fig. 4a, b and Supplementary Fig. 5e).

Pathway enrichment by gene set variation analysis was performed at the same time as the previous flow analysis on day 5 after adoptive transferred. In the oncolytic virus combined with adoptive T-cell therapy group, both OT-I and P14 cells showed enrichment for cytokine activity, the granzyme-mediated cell death pathway, and positive regulation of T-cell proliferation. Notably, combination treatment resulted in pathway enrichment in granzyme-mediated cell death in P14 CD8^+^ T cells. (Fig. 4c, d). The expression of various inhibitory receptors and transcription factors, such as Tox, Slamf6, Egr2, and Eomes, known to be associated with T-cell exhaustion, was found to be downregulated in OT-I cells from mice that received combination therapy of rVSV-LCMVG and T cells, compared to the cells isolated from the monotherapy group. In contrast, there was an upregulation in the expression of genes encoding effector molecules and inflammatory cytokine receptors, including Gzmb, Gzmk, Gzma, Ccr5, Ifng, and Stat1, in mice receiving the combination therapy. (Fig. 4e, f). Furthermore, the combined therapy not only reversed the exhausted phenotype of tumor antigen-specific T cells OT-I but also amplified the antitumor effects by enhancing the production of cytokines by virus-specific T cells (Fig. 4g, h). The study findings indicated that both OT-I and P14 T cells treated with the combination therapy exhibited a reduction in exhaustion signature, while demonstrating an increase in effector signatures.

### Transcriptional profiling of antitumor T cells in TME by scRNA-seq

**Fig. 5:**
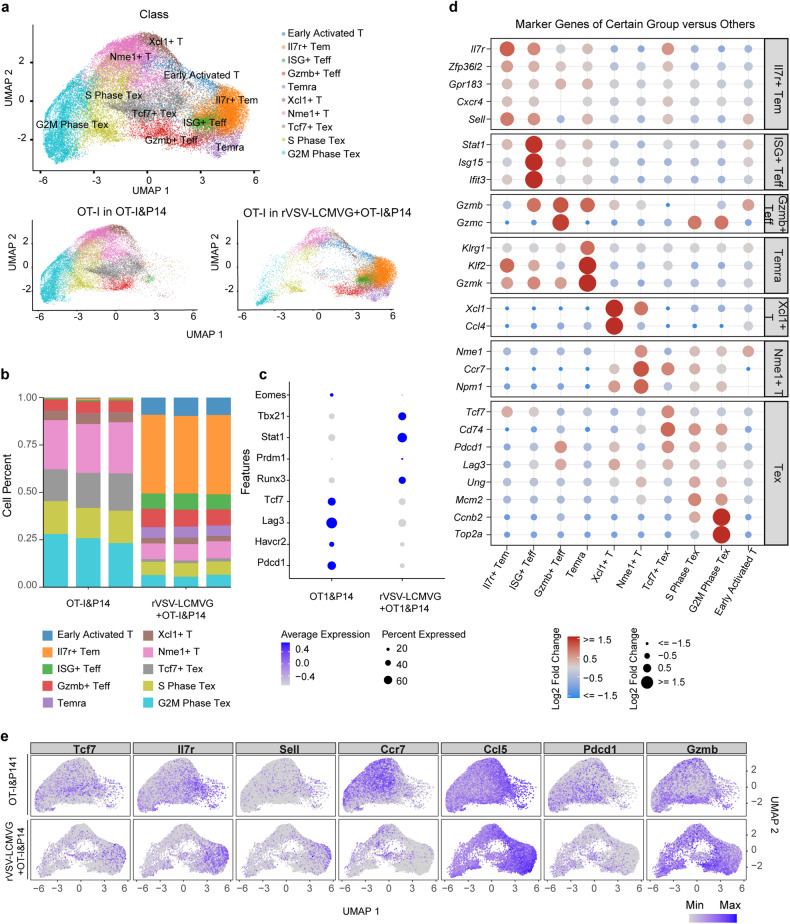
Transcriptional profiling of OT-I tumor-specific CD8^+^ T cells using scRNA-seq. **a** Uniform manifold approximation and projection (UMAP) visualization of the scRNA-seq clusters of OT-I tumor-specific CD8^+^ T cells from 6 samples in different groups. **b** Bar plot demonstrating percentages of cells in clusters as a fraction of total cells for each sample, related to the UMAP plot in **a**. **c** Dot plot representing the relative average expression of a subset of marker genes of OT-I tumor-specific CD8^+^ T cells in different groups. **d** Dot plot representing the relative average expression of a subset of marker genes across all clusters. **e** Single-cell transcription levels of representative genes illustrated in the UMAP plot from **a**. Transcription levels are color coded: gray, not expressed; blue, expressed

To gain a deeper understanding of how the differentiation process of tumor-specific and virus-specific T cells was affected by rVSV-LCMVG, we conducted single-cell RNA sequencing (scRNA-seq) analysis on these T cells following various in vivo treatments. We focused specifically on tumor-specific OT-I T cells obtained from B16-OVA engrafted C57BL/6 J mice. These T cells were then categorized into ten major clusters based on their characteristics: early activated T cells, Xcl1^+^ T cells, Il7r^+^ Tem cells, Nme1^+^ T cells, ISG^+^ Teff cells, Tcf7^+^ Tex cells, Gzmb^+^ Teff cells, S phase Tex cells, Temra cells, and G2m phase Tex cells. (Fig. 5a). OT-I T cells without rVSV-LCMVG stimulation were primarily found in exhausted T-cell clusters (G2m phase Tex, S phase Tex, and Tcf7^+^ Tex). However, when the TME was remodeled by rVSV-LCMVG, OT-I T cells predominantly belonged to effector T-cell clusters, including early activated T cells, ISG^+^ Teff cells, Temra cells, and Il7r^+^ Tem cells (Fig. 5b). In addition, OT-I cells exhibited elevated expression of Runx3 following treatment with combined OVs. This indicates that these OT-I cells may persist in tumor tissues for an extended duration, thereby exerting antitumor effects (Fig. 5c). Consistent with our previous findings, the administration of rVSV-LCMVG ameliorated the exhaustion phenotype of tumor-specific T cells by promoting the differentiation of Tex into effector T cells.

Next, we further validated the differentially expressed gene patterns of clusters that were significantly perturbed by rVSV-LCMVG treatment. G2M phase Tex expressed canonical exhaustion-related genes (Pdcd1, Ung, Mcm2, Ccnb2, and Top2a). Tcf7^+^ Tex was identified as the proliferative progenitor of terminally exhausted T cells. Nme1^+^ T cells expressing Nme1, Ccr7 and Npm1, were highly connected to Tcf7^+^ Tex cells. ISG^+^ effector T cells were further categorized based on Stat1, Isg15, Ifit3, and Gzmb expression. Il7r^+^ Tem highly expresses the signature of memory T cells (Il7r, Zfp36l2, Gpr183, Cxcr4, and Sell). Taken together, rVSV-LCMVG administration promotes tumor-specific exhausted (Tex) differentiation into effector (Teff) and memory (Tmem) cells with a significant decline in Tex proportion (Fig. 5d, e).

When analyzing virus-specific T cells, we observed that all samples could be classified into 13 distinct clusters. These clusters include early activated T cells, G2m phase Tex cells, Gzmb^+^ Teff cells, Il7r^+^ Tem cells, ISG^+^ Teff cells, ISG^+^ Bystander cells, Naïve-like T cells, Nme1^+^ T cells, S phase Tex cells, Regulator-like CD8 cells, Tcf7^+^ Tex cells, Xcl1^+^ T cells, and Terminally Tem cells. (Supplementary Fig. 6a, b). Combined with OVs, P14 virus-specific T cells differentiate from naive T cells into Teff and Tmem cells. In contrast, tumor-specific T cells undergo differentiation from Tex to Teff and Tmem cells (Supplementary Fig. 6c). This indicated that the adoptive transfer of tumor-specific T cells alone resulted in their differentiation into exhausted and disabled T cells upon tumor infiltration. However, when tumor-specific T cells were used in combination with the oncolytic virus rVSV-LCMVG, they effectively reversed exhaustion and improved their antitumor ability.

### mRNA tumor vaccine combined with oncolytic virus improved the therapeutic effect of B16 tumor

Given the high cost and challenges associated with personalized CAR T or TCR-T treatment, the induction of specific T cells through mRNA vaccines holds the potential to establish a more transformative therapeutic strategy. In this study, we explored the possibility of indirectly inducing tumor-specific T cells to replace the direct reinfusion of T cells. Instead of transferring P14 cells, we employed LCMV-Armstrong virus to induce specific T cells that recognize the gp33 epitope. Subsequently, we detected a certain proportion of these specific T cells in the peripheral blood, spleen, and lymph nodes of the abdominal groove. (Supplementary Fig. 7a, b). In the B16-GP33 model, we utilized LCMV-Armstrong immune-induced specific T cells along with the rVSV-LCMVG oncolytic virus, this combined approach demonstrated a notable efficacy in inhibiting tumor growth. It is important to highlight that the treatment effect was significantly superior to that of using the oncolytic virus alone. Furthermore, the combination therapy also led to a noticeable extension in the survival rate of mice as compared to the monotherapy treatment involving immune LCMV-Armstrong. (Supplementary Fig. 7c–e). In the B16-GP33 tumor model, immune LCMV-Armstrong effectively generated GP33-specific T cells, which successfully suppressed tumor growth. Then, we applied the same treatment strategies in the B16-OVA model to validate the results. The findings demonstrated that GP33-specific T cells induced by LCMV-Armstrong, which solely targeted the antigens carried by rVSV-LCMVG OVs and did not recognize tumor-associated antigens, when combined with rVSV-LCMVG could effectively restrain the growth of B16-OVA tumors and significantly prolonged the survival of mice. (Supplementary Fig. 7f–h). These results suggested that in addition to tumor-specific T cells, in the combination therapy using virus-specific T cells could also achieve better therapeutic effects.

**Fig. 6:**
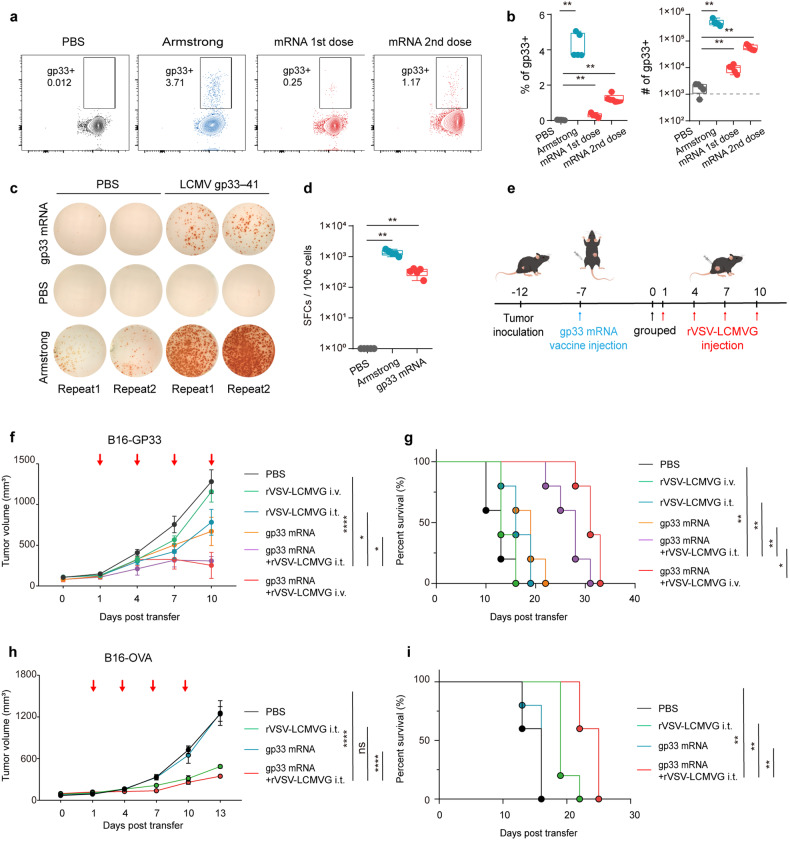
mRNA tumor vaccine combined with oncolytic virus improved the therapeutic effect. **a** Representative flow cytometry plot showing the fraction of gp33-specific T cells in the total CD8^+^ T cells gate from the spleen. **b** Quantification of the gp33-specific T cells. The proportion of cells in CD8 (left) and the total cell number (right). Each dot represents one mouse. **c** Representative well images of the IFN-γ ELISpot response of the gp33-specific T cells isolated from spleen in different groups. **d** Numbers of IFN-γ SFCs (spot-forming cells) of the gp33-specific T cells isolated from spleen were quantified after stimulation with GP33-41 peptide. **e** Schematic of B16-GP33 or B16-OVA tumor-bearing mice treated with gp33-mRNA and rVSV-LCMVG. **f** B16-GP33 tumor volumes are shown as mean values with SEM. Tumor response data derived from mice (*n* = 5) are shown. *p < 0.05; **p < 0.01; ***p < 0.001; ****p < 0.0001, based on two-way ANOVA with post hoc Holm–Sidak test. **g** Survival curves of C57BL/6 J mice from the experiment described in **f** are shown; survival analysis was conducted by log rank test. **h** B16-OVA tumor volumes are shown as mean values with SEM, (*n* = 5). *p < 0.05; **p < 0.01; ***p < 0.001; ****p < 0.0001, based on two-way ANOVA with post hoc Holm–Sidak test. **i** Survival curves of C57BL/6 J mice from the experiment described in **h** are shown; survival analysis was conducted by log rank test

Next, we prepared the mRNA tumor vaccine which could express gp33 epitope and we verified the expression of gp33 at the cellular level by immunofluorescence using an earlier G2B1 antibody that specifically recognizes the gp33 epitope (Supplementary Fig. 8a). Mice were immunized intramuscularly with a dose of 10 μg per mouse. The specific T cells capable of recognizing the gp33 epitope were identified seven days after immunization. After an interval of 14 days since the initial dose, the same vaccine dose was administered to enhance the immune response. Subsequently, after five days, an increase in the number of specific T cells in the spleen was observed (Fig. 6a, b). IFN-γ enzyme-linked immunosorbent spot (ELISpot) test results showed T cells from the immunized mice spleen had a strong response when stimulation with gp33-41 antigenic peptides ex vivo (Fig. 6c, d).

To determine the efficacy of mRNA vaccines combined with OVs in eliminating established tumors in vivo, we administered subcutaneous injections of 2 × 10^6^ B16-GP33 cells per mouse. Once tumor formation was evident at the injection site, muscular immunization was conducted, with each mouse receiving a dose of 10 µg mRNA. Following a 7-day interval, oncolytic virus therapy was administered (Fig. 6e). The intratumoral injection of rVSV-LCMVG or mRNA vaccine monotherapy resulted in a moderate inhibition of tumor growth compared to the PBS group. Combination therapy with intratumoral or intravenous injection of rVSV-LCMVG in combination with mRNA vaccines largely improved the responsiveness of B16-GP33 tumors and prolonged the survival of these mice (Fig. 6f, g and Supplementary Fig. 8b). In combination therapy using mRNA vaccines, the therapeutic efficacy of the rVSV-LCMVG oncolytic virus was found to be superior in the intravenous injection group compared to the intratumoral administration. This may be attributed to the fact that, after immunization with mRNA vaccines, the intravenous administration of OVs stimulated a stronger systemic immune response than the intratumoral administration. As a result, there was an increase in the production of specific T cells and improved therapeutic outcomes. Even in the B16-OVA model, mRNA was only able to induce the generation of virus-specific T cells, also emphasizing that the combination therapy approach yielded better therapeutic results (Fig. 6h, i and Supplementary Fig. 8c). Our results highlight that while using mRNA to induce oncolytic virus-specific T cells or tumor-specific T cells, combined therapy with oncolytic virus would lead to a better therapeutic effect, especially when the mRNA-induced specific T cells could recognize both tumor and OVs, even if the oncolytic virus was administered intravenously, mice would gain a better therapeutic effect compared to monotherapy.

## Discussion

In our study, we utilized the G protein of LCMV to replace the G protein of VSV in order to construct the rVSV-LCMVG oncolytic virus. This modification strategy was employed to enhance the safety profile of VSV as an oncolytic virus for intravenous administration, thereby reducing the likelihood of generating neutralizing antibodies and enabling multiple administrations. We investigated the efficacy of combining rVSV-LCMVG with T-cell therapy and tumor mRNA vaccines for the treatment of B16 solid tumors, which serve as a murine model of highly immunosuppressive melanoma. Individually, the treatment effect of rVSV-LCMVG or tumor-specific OT-I T cells with virus-specific P14 T cells alone in the B16-OVA model was found to be insufficient. However, when these two treatments were combined, significant tumor suppression was observed, indicating a synergistic effect. Our findings demonstrated that the presence of adoptively transferred cells within the tumor was markedly increased upon rVSV-LCMVG treatment. Additionally, the functionality of tumor-infiltrating T cells was enhanced after rVSV-LCMVG administration. These results indicate that rVSV-LCMVG effectively overcame the highly immunosuppressive tumor immune microenvironment, leading to improved immune responses and tumor control. Furthermore, our study revealed that OVs have the ability to augment the antitumor effectiveness of tumor mRNA vaccines. Flow cytometry and single-cell RNA sequencing analyses presented compelling evidence indicating that OVs modify the immunosuppressive TME. This modulation leads to the differentiation of tumor-specific T cells into potent effector cells, rather than exhausted cells. These findings shed light on the potential mechanism underlying the enhanced therapeutic efficacy of T cells by OVs.

Malignant tumors have long posed a significant threat to human life and well-being. Currently, immunotherapy has emerged as a new ray of hope in the battle against cancer, with CAR-T therapy standing out as one of the most promising tumor immunotherapies in recent years. However, it is important to note that most of the successful immune cell therapies available on the market are primarily effective for hematological tumors. There remain significant challenges in effectively combating solid tumors, which represent the “main battlefield” in the fight against cancer. In the present study, we have successfully demonstrated that OVs have the potential to enhance the therapeutic efficacy of T-cell-based therapies for solid tumors. This combination approach addresses the limitations associated with individual oncolytic virus or T-cell therapies. In addition to our research, several other research groups have also reported the synergistic effect of T-cell therapy and OVs in solid tumors through diverse in vivo and in vitro experiments.^34–36^ However, in their studies the underlying mechanisms driving this synergy remain largely unknown and warrant further investigation.

To investigate the specific changes induced by OVs on tumor-specific T cells and virus-specific T cells, we employed a combination therapy approach utilizing OVs and adoptively transferred T-cell therapy in model antigen-carrying tumor models. Notably, the combination of T cells and OVs led to a significant improvement in T-cell infiltration into tumors. Moreover, this combination therapy resulted in a reduction in the expression of immunoregulatory genes such as Pdcd1, Havcr2, and Lag3, indicating a favorable modulation of the TME. Activation of the GZM-mediated tumor cell death pathway was also observed, resulting in enhanced tumor-killing capabilities of the T cells. Furthermore, the expression level of Runx3, a marker associated with tissue-resident characteristics, was significantly upregulated following the administration of OVs. This upregulation allows T cells to reside within the TME and exert their therapeutic effects for a longer time. These molecular-level insights further confirm the ability of OVs to promote the tumor treatment efficacy of T cells and shed light on the underlying mechanisms by which OVs enhance the therapeutic effects of T cells.

In order to overcome the challenges associated with the high cost and technical complexity of individualized CAR-T or TCR-T-cell therapies, we explored the use of mRNA vaccines to induce specific T-cell responses. Building on our previous experience with T-cell combination therapies, we ventured into uncharted territory by combining mRNA vaccines with OVs for tumor treatment. Notably, we were surprised to observe a remarkable efficacy of this combination therapy when administered intravenously. We hypothesized that this enhanced efficacy may be attributed to the systemic immune enhancement triggered by the intravenous administration of OVs following the use of tumor mRNA vaccines, as opposed to the local immunity promoted by intratumoral administration of OVs. The combined approach presented in this study effectively tackles the limitations associated with systemic delivery of OVs, resulting in a substantial enhancement in the use of these OVs for therapeutic purposes.

In addition to investigating its effects on the immune system in the TME. We believe that understanding the impact of rVSV-LCMVG on tumor immunogenic cell death could also provide valuable insights into the potential bridge between oncolytic effects and immune regulation. This will enable us to better elucidate the mechanism through which the combination of adoptively transferred T cells and mRNA vaccines, along with rVSV-LCMVG, can achieve enhanced antitumor effects compared to monotherapy. The present study aimed to investigate the therapeutic effect and mechanism of OVs when combined with specific T cells or mRNA. And in this paper we only focused on utilizing tumor models such as B16-GP33 or B16-OVA, which possess specific epitopes, as well as the widely recognized immunological research tools like P14 and OT-I cells. However, our study did not concentrate on elucidating the precise pathways and pivotal molecules involved in the therapeutic actions, and it lacked validation in a broader range of tumor models. In future endeavors, we intend to substitute the artificially added epitope with tumor neoantigens, utilizing mRNA technology, to explore the therapeutic efficacy of oncolytic virus combined with tumor neoantigen vaccine specifically in different solid tumors.

In conclusion, our study demonstrated that the combination therapy with oncolytic virus and adoptively transferred T cells could amplify their antitumor effects, we also shed light on the mechanisms through which OVs enhance the antitumor efficacy of adoptively transferred T cells, which might provide valuable insights into its potential clinical applications. Notably, we proposed that the administration of mRNA vaccines in combination with OVs holds promise as an effective therapeutic strategy for solid tumors.

## Materials and methods

### Animals

P14 animals with a transgenic TCR recognizing LCMV glycoprotein 33 (gp33-41) and OT-I animals with a transgenic TCR recognizing ovalbumin (OVA257–264) peptide were used for in the experiments. P14-TCR transgenic mice and OT-I TCR transgenic mice were maintained on a C57BL/6 J background, P14 mice were backcrossed to Thy1.1^+^ (CD90.1). OT-I animals were backcrossed to CD45.1^+^ and bred at the Laboratory Animal Center of Xiamen University. For all studies, age-matched and sex-matched mice (age: 6–12 weeks) were used. They were purchased from the Shanghai SLAC Laboratory Animal Co., Ltd. Mice of the same sex were randomly assigned to an experimental group. In terms of tumor burden, all experimental groups had similar mean tumor volumes before treatment. All mice were maintained under specific pathogen-free conditions at the Laboratory Animal Center of Xiamen University.

All experimental procedures with mice were performed with the approval of the Institutional Animal Care and Use Committee of Xiamen University (XMULAC20240079), and in accordance with the Guide for the Care and Use of Laboratory Animals.

### Cell lines

A549, H1703, H1299, and some other cells were purchased from ATCC. EBC-1, HepG2, BEL-7704 and some other cells were derived from pre-preserved cells in our laboratory National Institute of Diagnostics and Vaccine Development in lnfectious Diseases (Xiamen University) (NIDVD). This form provides detailed information regarding the source of each cell line as well as the optimal culture conditions.

**Table:** 

Cell lines	Source	Medium
A549	ATCC	DMEM + 10%FBS
H1703	ATCC	1640 + 10%FBS
H1299	ATCC	DMEM + 10%FBS
EBC-1	NIDVD	DMEM + 10%FBS
H661	ATCC	1640 + 10%FBS
SMMCC7721	NIDVD	DMEM + 10%FBS
HUH7	ATCC	DMEM + 10%FBS
HepG2	NIDVD	DMEM + 10%FBS
BEL-7704	NIDVD	DMEM + 10%FBS
BEL-7702	NIDVD	DMEM + 10%FBS
QSQ-7701	NIDVD	DMEM + 10%FBS
QGY-7703	NIDVD	DMEM + 10%FBS
Hep1-6	ATCC	DMEM + 10%FBS
Hep3B	ATCC	DMEM + 10%FBS
PLC/PRF/5	ATCC	DMEM + 10%FBS
AGS	ATCC	DMEM + 10%FBS
SCG7901	ATCC	DMEM + 10%FBS
BGC823	NIDVD	DMEM + 10%FBS
CT26	ATCC	1640 + 10%FBS
MC38	NIDVD	DMEM + 10%FBS
HCT116	ATCC	DMEM + 10%FBS
SW1116	NIDVD	DMEM + 10%FBS
SW480	CTCC	DMEM + 10%FBS
B16	NIDVD	DMEM + 10%FBS
SIHA	ATCC	DMEM + 10%FBS
CASKI	ATCC	DMEM + 10%FBS
HELA	NIDVD	DMEM + 10%FBS
ES-2	ATCC	DMEM + 10%FBS
A2870	ATCC	DMEM + 10%FBS
SKOV-3	ATCC	DMEM + 10%FBS
CFPAC-1	ATCC	DMEM + 10%FBS
panc-1	NIDVD	DMEM + 10%FBS
SW1990	ATCC	DMEM + 10%FBS
ASPC-1	ATCC	1640 + 10%FBS
HPAF-II	ATCC	DMEM + 10%FBS
Panc 02.03	ATCC	1640 + 10%FBS
Panc 10.05	ATCC	1640 + 10%FBS
BxPc-3	ATCC	DMEM + 10%FBS
MIA-PA-CA	NIDVD	DMEM + 10%FBS
ZR-75-30	NIDVD	DMEM + 10%FBS
CNE-1	ATCC	DMEM + 10%FBS
U87	ATCC	DMEM + 10%FBS
SHSY5Y	ATCC	DMEM + 10%FBS
G422	ATCC	DMEM + 10%FBS
GL261	ATCC	DMEM + 10%FBS
MCF7	NIDVD	DMEM + 10%FBS
4T1	ATCC	1640 + 10%FBS
MDA-MB-453	ATCC	DMEM + 10%FBS
Hep2	ATCC	DMEM + 10%FBS
U2OS	ATCC	DMEM + 10%FBS
DU145	ATCC	DMEM + 10%FBS
293B5	NIDVD	DMEM + 10%FBS
HPNE	NIDVD	DMEM + 10%FBS
LO2	ATCC	DMEM + 10%FBS
B16-GP33	NIDVD	DMEM + 10%FBS
B16-OVA	NIDVD	DMEM + 10%FBS

### Oncolytic virus rVSV-LCMVG production

Oncolytic virus rVSV-LCMVG was generated by replacing the VSV glycoprotein with the LCMV glycoprotein gene which was codon-optimized for expression in human cells.^37^ Firstly, 293 T cells were plated on poly-L-lysine solution (Sigma-Aldrich) treated plates and incubated overnight in DMEM (Sigma-Aldrich) containing 10% FBS (Gibco) and 1% penicillin/streptomycin/L-glutamine (Invitrogen). The following day, cells were infected by recombinant vaccinia virus producing the T7 RNA polymerase (rVV-T7) and transfected with the VSV genomic clone driven by a T7 promoter and helper plasmids expressing the VSV-N, VSV-P, VSV-M, LCMVG, and VSV-L with lipofectamine LTX reagent (Invitrogen). After 48 h, the supernatants of the transfected cells were co-cultured with Vero E6 cells (ATCC). Cells were monitored for cytopathic effect indicative of virus replication. Viruses were then expanded and titrated in BHK21 cells, then viruses were clarified by centrifugation at 3500 rpm for 5 min and frozen at −80 °C until use. The titer of the recombinant virus was determined using a plaque assay.

### Tumor models

Healthy naive female C57BL/6 J mice (age: 6–8 weeks) were purchased from Shanghai SLAC Laboratory Animal Co., Ltd. All mice were maintained under specific pathogen-free conditions at the Laboratory Animal Center of Xiamen University. Mice were placed in designated groups, and their status (e.g., tumor size) was monitored. The mice were immediately euthanized when ethical endpoints were reached.

C57BL/6 J mice were injected with B16-OVA or B16-GP33 melanoma cell line 5 × 10^6^ cells in 100 μL of PBS subcutaneously. Once the tumors reached approximately 100 mm^3^, the activated P14 and OT-I cells were intravenously injected into the caudal vein of the tumor-bearing mice

One day later the rVSV-LCMVG OVs was intratumorally injected at a dose of 1 × 10^7^ PFU every 3 days until a total of four dosages were completed. Tumor volumes were measured every 3 days using calipers and calculated using the following formula: volume = (length × width^2^)/2. For analytical experiments, to get enough cells for analysis, the mice were treated with OVs only two dosages, then euthanized and the tumors were harvested and processed for flow cytometry. For all studies, the mice were euthanized once the tumors reached a diameter of 15 mm.

### Supplementary information

Supplemental material

## Data Availability

All the datasets presented in the paper are available from the corresponding author upon reasonable request.

## Code availability

The raw and processed data of RNA-seq and scRNA-seq experiments generated in this study have been deposited in the Gene Expression Omnibus (GEO) repository under SuperSeries accession code GSE235291.
