# Supplementary material for: Combination therapy with oncolytic virus and T cells or mRNA vaccine amplifies antitumor effects
Source: Signal Transduct Target Ther. 2024 May 3;9:118. doi: 10.1038/s41392-024-01824-1 (PMC11068743; doi:10.1038/s41392-024-01824-1)
Supplement: Supplementary file 1 — Supplemental material [file 41392_2024_1824_MOESM1_ESM.docx]

Supplementary Materials for

Combination therapy with oncolytic virus and T cells or mRNA vaccine amplifies antitumor effects

Rao Fu,†, Ruoyao Qi,†, Hualong Xiong,†, Xing Lei,†, Yao Jiang,†, Jinhang He, Feng Chen, Liang Zhang, Dekui Qiu, Yiyi Chen, Meifeng Nie, Xueran Guo, Yuhe Zhu, Jinlei Zhang, Mingxi Yue, Jiali Cao, Guosong Wang, Yuqiong Que, Mujing Fang, Yingbin Wang, Yixin Chen, Tong Cheng, Shengxiang Ge, Jun Zhang, Quan Yuan*, Tianying Zhang*, Ningshao Xia*

† These authors contributed equally to this work: Rao Fu, Ruoyao Qi, Hualong Xiong, Xing Lei, Yao Jiang

*Correspondence to: Quan Yuan (yuanquan@xmu.edu.cn) or Tianying Zhang (zhangtianying@xmu.edu.cn) or Ningshao Xia (nsxia@xmu.edu.cn)

**This file includes:**

Materials and Methods

Figures. S1 to S8

**Materials and Methods**

**Quantitative PCR analysis for VSV RNA**

Total RNA was extracted from serum or tissues by GenMagbeads Viral DNA/RNA Kit (NA007-3, genmagbio) according to the manufacturer's instructions. Quantitative PCR analysis for VSV RNA was performed using LightCycler Systems (Roche). The probe consists of forward primers that were synthesized by Sangon (5'- CTTCCAGATGGAGTATCGGATG-3') and (reverse primers, 5' -gTgTTCTgCCCACTCTgTATAA-3') combined with a 5' -FAM-AAggCAACCATTTgTCATCTgCgC-BHQ-3' –labeled probe specific for the N gene of VSV. To determine RNA copy numbers, a standard curve was run in parallel with each analysis using the serial log-fold dilutions of VSV genome plasmid pVSV eGFP dG (#31842, Addgene) in nuclease-free water, and concentrations of VSV RNA in each PCR reaction were expressed as copies of VSV N gene. The total amounts of VSV RNA in tissues (copies/100mg tissue) were calculated using the following formula: N RNA content/(100mg tissue weight).

**In vitro cell cytotoxicity assay**

The cytotoxicity caused by rVSV-LCMVG on cells were measured by CCK-8 reagent according to the manufacturer’s protocols. Cells were seeded and cultured at a density of 3 × 10^4^/well in 100 μL of medium containing 10% FBS into 96-well microplates (Corning) and infected with rVSV-LCMVG at different MOIs. After treatment for 48h, the CCK-8 reagent (10μL) was added to 90μL DMEM to generate a working solution, of which 100μL was added per well and incubated for 1.5h. All experiments were performed in duplicate. The absorbance was measured at a wavelength of 450nm using a microplate spectrophotometer (Thermo Fisher Scientific). IC50 value was determined by the nonlinear regression analysis using GraphPad Prism 9.4.0.

**In vitro cultures**

The B16-OVA mouse melanoma cell line expressing the ovalbumin protein and the B16-GP33 mouse melanoma cell line expressing LCMV glycoprotein 33 were cultured in DMEM supplemented with 10% (v/v) FBS (Invitrogen), containing 1% penicillin/streptomycin. All cells were maintained at 37°C and 5% CO_2_. For lymphocyte harvest, sorting, and in vitro restimulation, mice were sacrificed at specific time points, and cell suspensions were prepared from spleens, draining lymph nodes, and tumors. Tumors were dissected, transferred to 1640 medium supplemented with DNase I (200 μg/mL; Roche) and Collagenase IV (100 μg/mL; GIBCO), and incubated at 37 °C for 1 h. Tissue suspensions were passed through 70-μm strainers, washed with PBS containing 2% FBS, and treated with Ficoll-Paque Premium 1.084 separation reagent to obtain lymphocytes.

For cytokine analysis upon ex vivo restimulation, cell suspensions were plated in 96-well plates before adding 4 μg/mL cognate peptides (OVA257–264 and LCMV gp33–41, gp61-75, and gp276-286). Brefeldin A was added after incubation for 1 h at 37 °C. After an additional 4 h at 37 °C, cells were collected, stained with surface markers, and fixed with Fixation for 40 min at 4 °C. Permeabilization and cytokine staining were performed. Data were acquired on Fortessa and analyzed using FlowJo software.

**Immunofluorescence microscopy**

B16-OVA cells (4 × 10^4^) were plated in individual wells of 96-well glass-bottom plates (CellCarrier-96 Black) and incubated for 12 h to allow cells to adhere. Subsequently, B16-OVA cells were infected with rVSV-LCMVG for 16 h at varying MOI. Media were abandoned from each well and the cells were gently washed with PBS. The cells were then fixed and permeabilized for 10 min at room temperature. Then stained with anti-LCMVG (GP33) antibody generated by our laboratory (1:200) and anti-VSV antibody generated by our laboratory (1:200) for 1 h at room temperature, then washed, and stained with goat anti-mouse IgG Alexa Fluor 488 or 647(Abcam) for 1 h. The cells were then stained with 4′6-diamidino-2- phenylindole (DAPI) for 5 min at room temperature, and each image was captured using Opera Phenix.

**OV transduction and T cell functional assays in vitro**

For rVSV-LCMVG transduction and cells killing assays, B16-OVA cells infected rVSV-LCMVG for 1 h, and then co-cultured with P14 cells at effector T cell to tumor cell ratios of 1:1 for 0, 12, and 18 h, and visualized via phase-contrast microscopy. The supernatant and cells were also collected to detect the concentration of IFN-γ or analyzed by flow cytometry for specific markers of T cell activation.

**Activation of CD8+ T cells**

To obtain OT-I and P14 cells, total CD8+ T cells were harvested from the spleen and lymph nodes of 6- to 10-week-old P14 or OT-I mice, then disrupted in PBS containing 2% fetal bovine serum (FBS). Fragments were removed by passing the cell suspension through a 70-μm mesh nylon strainer. The cells were centrifuged at 300 × *g* for 5 min and resuspended at 1 × 10^8^ cells/mL in PBS containing 2% FBS and 1 mM EDTA. Next, the OT-I and P14 cells were negatively selected using the EasySep™ Mouse CD8+ T Cell Isolation Kit (Stemcell).

For OT-I and P14 cell activation with anti-CD3/CD28 beads or anti-CD3 (clone 2C11) plus anti-CD28 (clone 37.51), 2 × 10^7^ OT-I or P14 cells isolated from spleen and lymph nodes were cultured in 175 cm^2^ tissue culture flasks within 20 ml of T cell media (RPMI Medium 1640 with 10% heat inactivated FBS, 2 mM L-Glutamine, 100 U/mL penicillin/streptomycin and 50 mM beta-mercaptoethanol, 50 units/mL murine recombinant IL-2) with 2 × 10^7^ anti-mouse CD3/CD28 beads or anti-CD3 (clone 2C11) plus anti-CD28 (clone 37.51) pre-coated plates. All cells were incubated in a humidified CO_2_ incubator at 37°C for two or three days. After removing the beads, the activated T cells were harvested for transferring.

**RNA-Seq**

OT-I and P14 cells were sorted on BD FUSION Sorters from tumor-bearing animals that were treated with rVSV-LCMVG combined with T cells or T cells alone, and the sorted cells were resuspended in TRIzol and frozen. Samples were shipped to OE Biotech for RNA isolation, library preparation, and RNA sequencing.

**scRNA-Seq**

The same cell sorting strategy was used for single-cell RNA sequencing. Single-cell suspensions were loaded into microfluidic devices, and scRNA-seq libraries were constructed according to the Singleron GEXSCOPER protocol using the GEXSCOPER Single-Cell RNA Library Kit (Singleron Biotechnologies, Jiangsu, China)


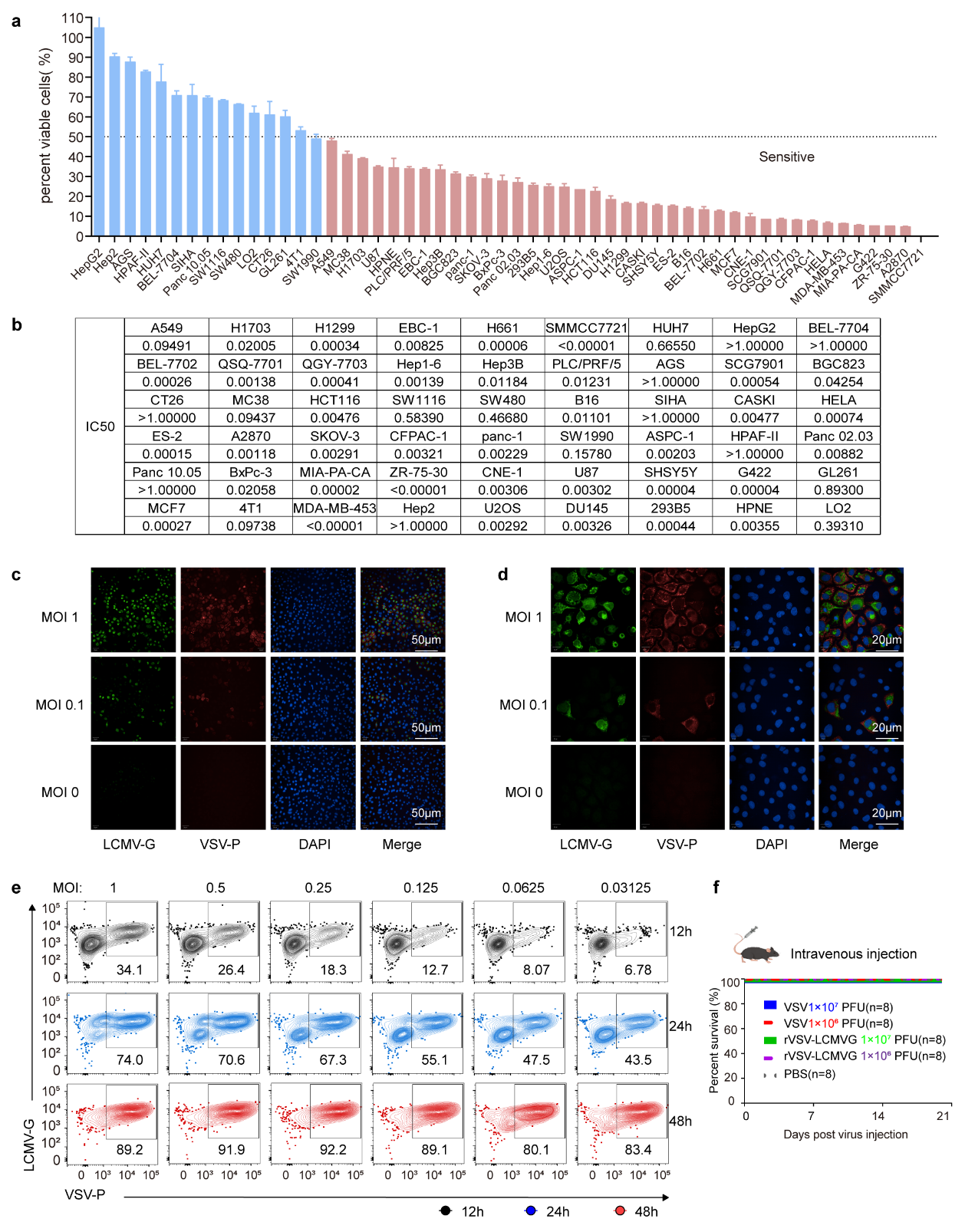


**Figure. S1. Characterization of rVSV-LCMVG.**

**a.** Cell viability of total 54 cancer cell lines after treatment with 0.1 MOI rVSV-LCMVG for 48 hours. **b**. IC50 of rVSV-LCMVG for cancer various cell lines. **c, d.** Immunofluorescence microscopy of B16-OVA cells infected for 16 hours with rVSV- LCMVG at MOI of 0 (uninfected), 0.1, or 1. Blue, DAPI; red, VSV P protein; green, LCMV-G protein. Scale bars, 50 μm or 20 μm. **e**. B16-OVA tumor cells positive for G protein of LCMV and P protein of VSV after 12, 24, and 48 hours of rVSV-LCMVG infection at increasing MOIs. Percentage values indicate LCMV-G, VSV-P positive population in the boxed. **f**. Various doses (1×10^7^ PFU and 1×10^6^ PFU) of rVSV-LCMVG or wild-type virus VSV, inoculated by tail vein injection, to monitor survival of mice.


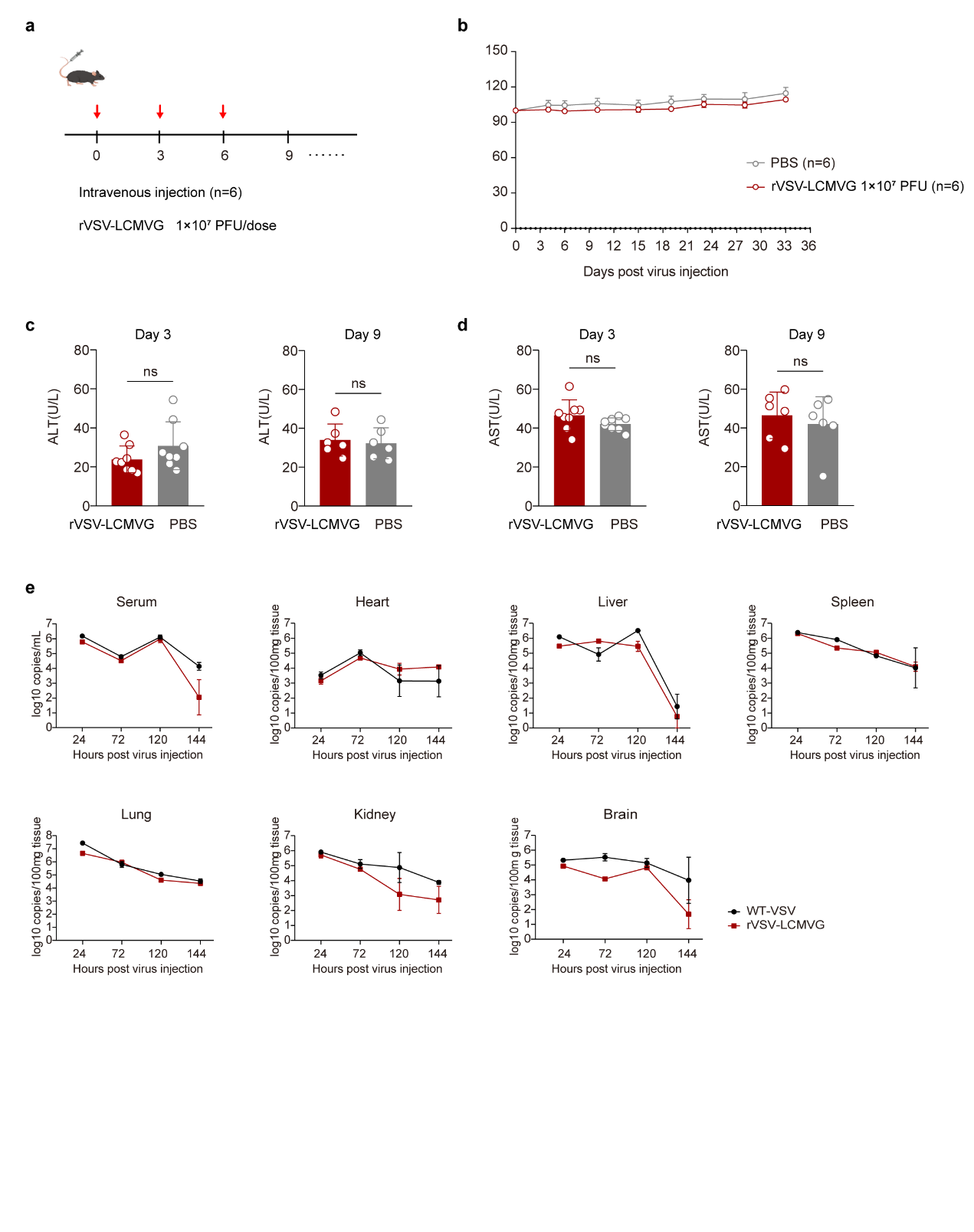


**Figure. S2. Assessment of the Safety of rVSV-LCMV Oncolytic Virus.**

**a**. Schematic of mice treated with rVSV-LCMV (1×10^7^ PFU/dose) by tail vein injection to monitor the weight. **b**. Weight changes of the mice postinjection. Data points represent means SDs of each cohort at the respective time-point (n=6 per group). **c, d**. ALT and AST were determined at the indicated time-points postinjection. Each dot represents one mouse, ns means not significant based on the Mann Whitney test. **e**. Biodistribution of rVSV-LCMVG in mouse serum and tissues. Mice received 1×10^7^ PFU WT-VSV or rVSV-LCMVG via i.v. injection, Concentration-time profiles were generated by measuring viral genomes by qRT-PCR of tissue homogenates collected at necropsy 24, 72, 120 or 144 h post-dose. Data points represent mean concentrations (±SEM) from n = 4 animals per time point.


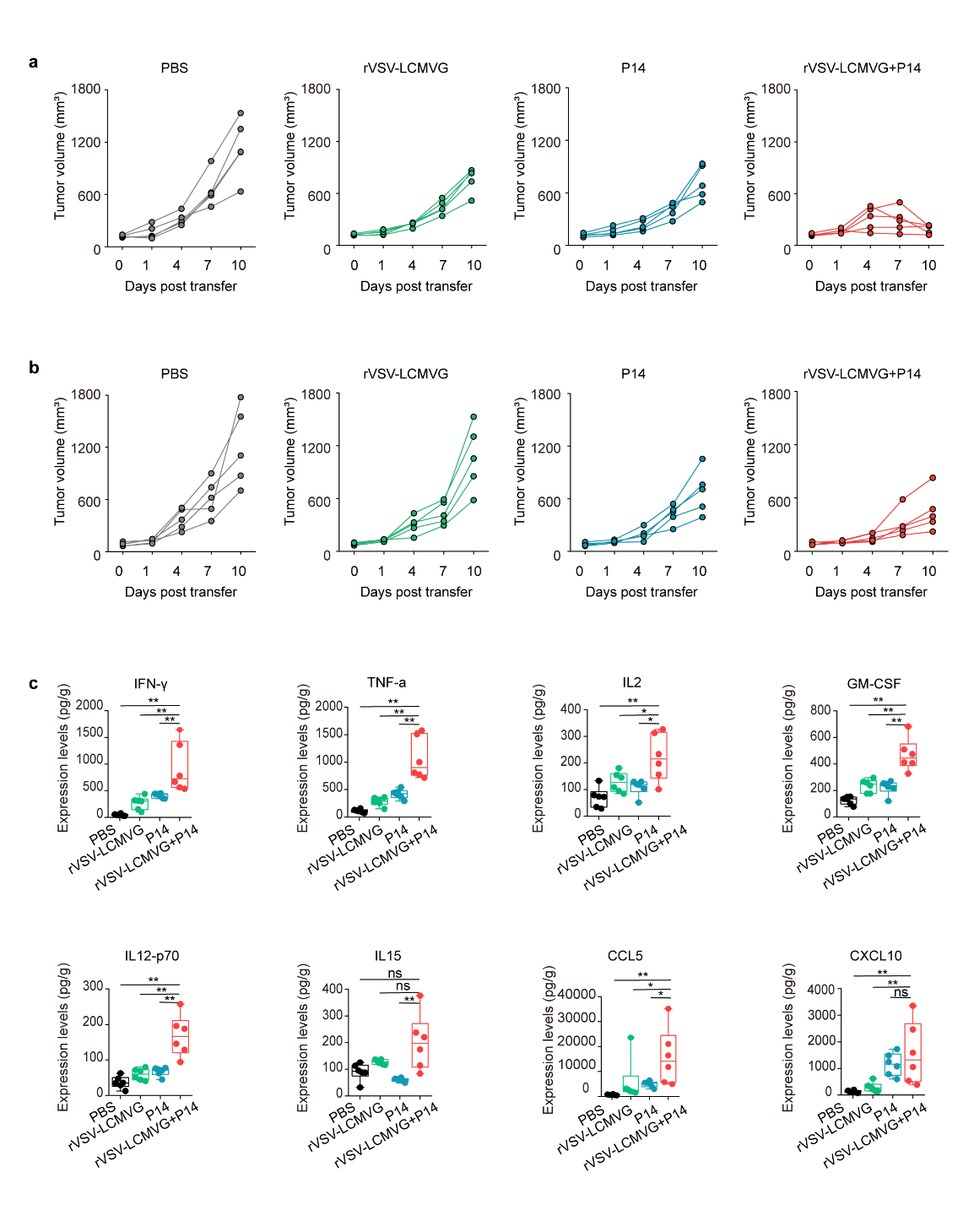


**Figure. S3.** **Antitumor efficacy of rVSV-LCMVG combined with P14 cells in B16-GP33 tumor models.**

**a**. Tumor volumes for each mouse in each treatment group are shown for mice described in (Figure 2**b**). **b**. Tumor volumes for each mouse in each treatment group are shown for mice described in (Figure 2**d**). **c**. Tumors from B16-GP33 tumor-bearing mice treated with PBS, rVSV-LCMVG, P14 T cells, and combination of rVSV-LCMVG and P14 T cells, 5 days after in vivo transfer, were assayed for cytokine and chemokine expression. Data are expressed as pg/g.


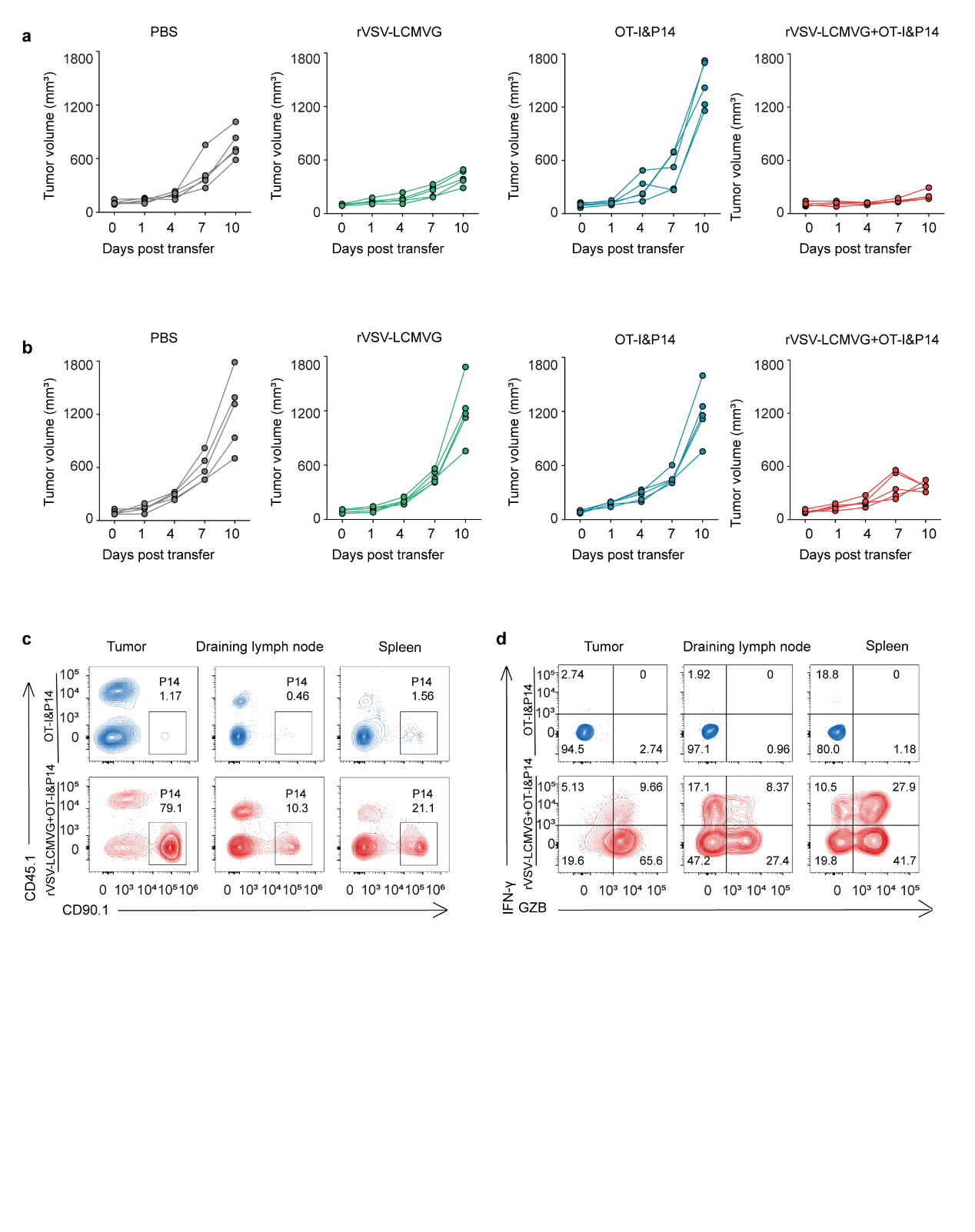


**Figure. S4. Antitumor efficacy of combination therapy of rVSV-LCMVG and P14, OT-I cells in B16-OVA tumor models.**

**a**. Tumor volumes for each mouse in each treatment group are shown for mice described in (Figure 3**b**). **b**. Tumor volumes for each mouse in each treatment group are shown for mice described in (Figure 3**d**). **c.** Flow cytometry plot showing the proportion of P14 (CD90.1+) cells in the total CD8+ T-cell gate, in the tumor, draining lymph node, or spleen of a representative mouse, 5 days after in vivo transfer. **d**. Representative intracellular staining for the cytokines IFN-γ and GZMB, upon restimulation of P14 cells isolated from the tumor (Left), draining lymph node (Middle), or spleen (Right) with 4 mg/ml LCMV-specific CD8 peptides (GP33-41, GP276-286) and 4 mg/ml CD4 peptide (GP61-80).


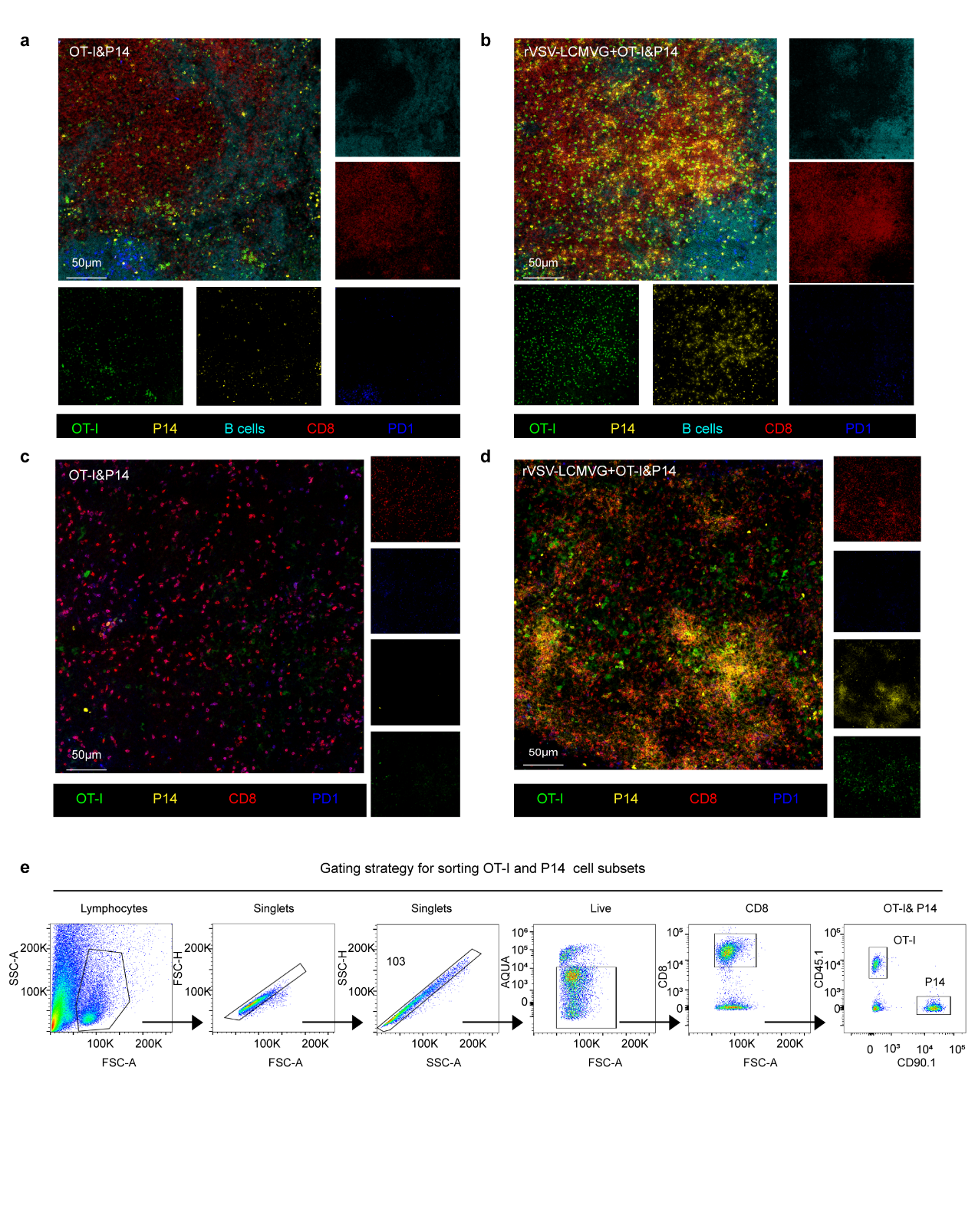


**Figure. S5. Analysis of Immune Cell Infiltration in Tumors or Lymph Nodes.**

**a**, **b**. Representative multiplexed immunofluorescence images of the draining lymph nodes in B16-OVA tumor model [rVSV-LCMVG-treated (**b**) and rVSV-LCMVG-nontreated (**a**)] combined with OT-I and P14, determined using multiplex immunofluorescence imaging, shows immune infiltration. **c, d**. Representative multiplexed immunofluorescence images of the tumor in B16-OVA tumor model [rVSV-LCMVG-treated (**d**) and rVSV-LCMVG-nontreated (**c**)] combined with OT-I and P14, determined using multiplex immunofluorescence imaging, shows immune infiltration. **e**. Gating strategy for OT-I and P14 T cells for RNA-seq in the tumor.


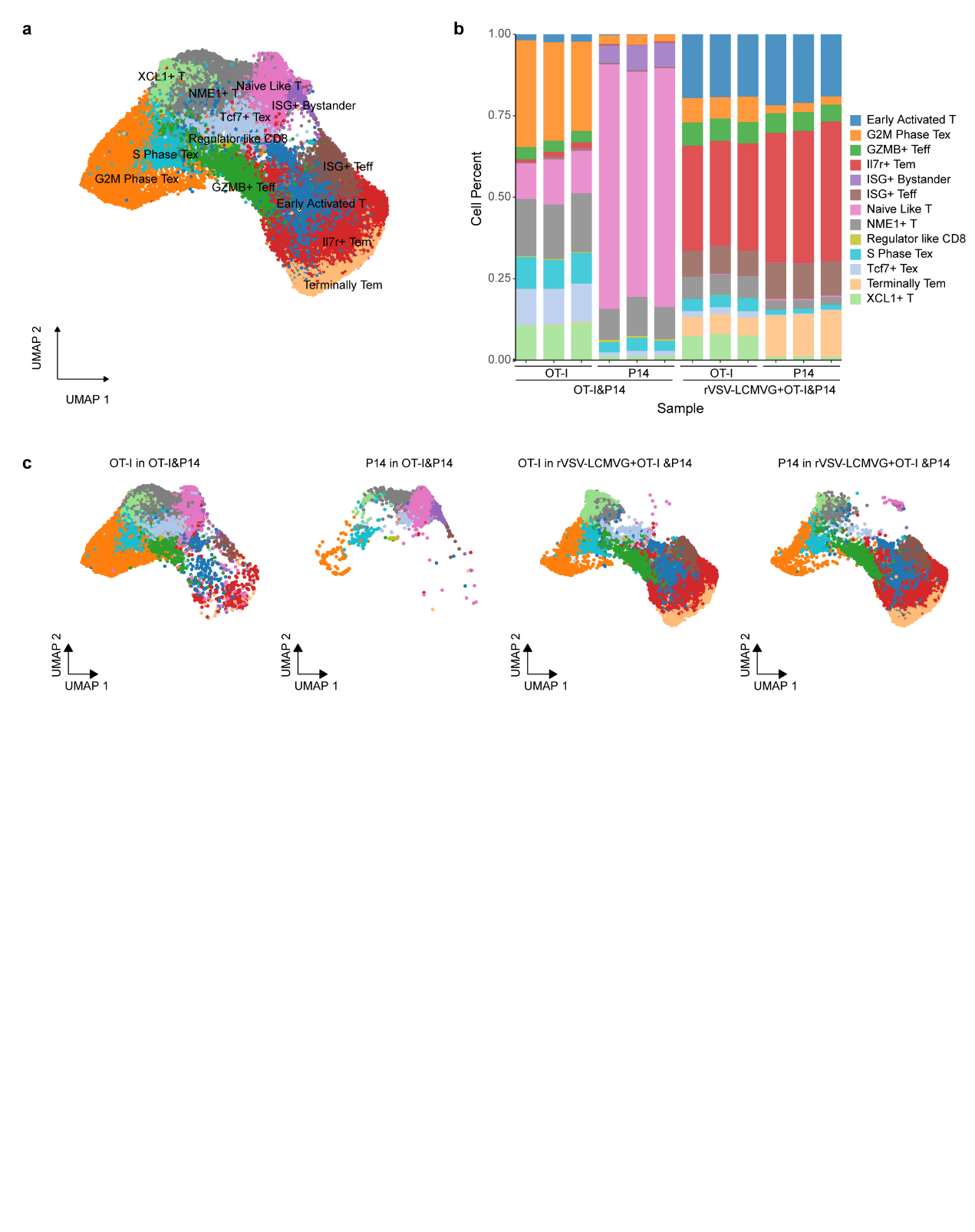


**Figure. S6. Transcriptional profiling of OT-I and P14 using scRNA-seq.**

**a.** Uniform manifold approximation and projection (UMAP) visualization of the scRNA-seq clusters of OT-I tumor-specific CD8+ T and P14 virus-specific T cells from 6 samples in different groups. **b**. Bar plot showing percentages of cells in clusters as a fraction of total cells for each sample, related to the UMAP plot in (**a**). **c**. Uniform manifold approximation and projection (UMAP) visualization of the scRNA-seq clusters of OT-I tumor-specific and P14 virus-specific T cells from each group


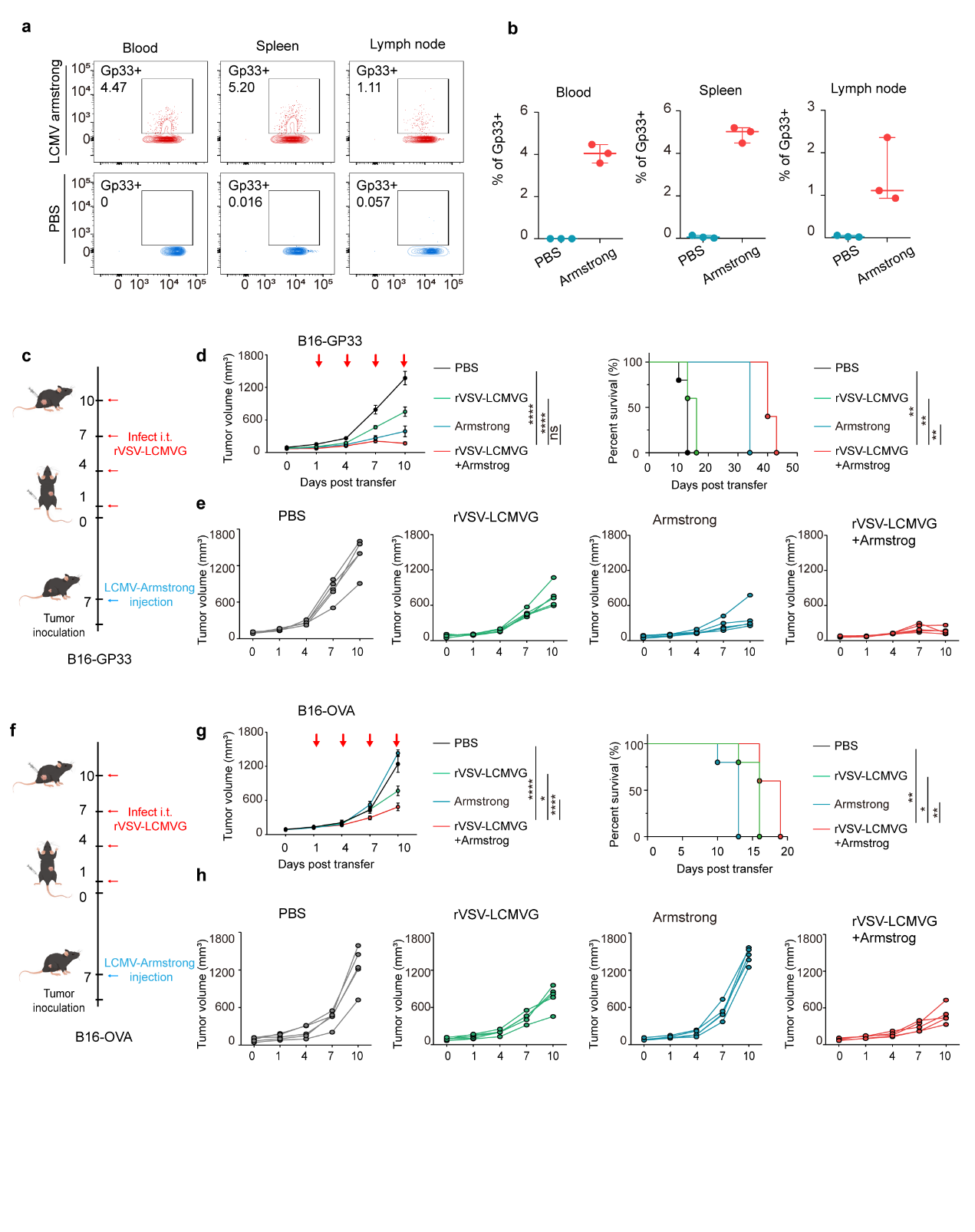


**Figure. S7.** **Combining Specific T Cells With Oncolytic Viruses In Tumor Treatment.**

**a.** Representative flow cytometry plot showing the proportion of gp33-specific T cells induced by intraperitoneal immunization of 1×10^5^ PFU LCMV-Armstrong virus in peripheral blood, spleen, and the lymph nodes of the abdominal groove. **b.** Quantification of the proportion of gp33-specific T cells in the total CD8+ T-cell gate in in the peripheral blood, spleen, and lymph nodes of the abdominal groove. Each dot represents one mouse. **c.** Schematic of B16-GP33 tumor-bearing mice treated with LCMV-Armstrong and rVSV-LCMVG. C57BL/6J mice were injected subcutaneously with 2 × 10^6^ B16-GP33 cells per mouse; when tumor formation could be observed at the inoculation site, LCMV-Armstrong virus intraperitoneal immunization was carried out a dose of 1 ×10^5^ PFU per mouse, and after 7 days, oncolytic virus rVSV-LCMVG was administered at 1 ×10^7^ PFU per dose, every 3 days, for consecutive 12 days. **d**. Tumor volumes are shown as mean values with SEM. Survival curves of C57BL/6J mice from the experiment described in (**c**) are shown. Tumor response data derived from mice (n = 5) are shown. *p < 0.05; **p < 0.01; ***p < 0.001; ****p < 0.0001, based on two-way ANOVA with post hoc Holm-Sidak test; survival analysis was conducted using log rank test. **e**. Tumor volumes for each mouse in each treatment group are shown for mice described in (**d**). **f**. Schematic of B16-OVA tumor-bearing mice treated with LCMV-Armstrong and rVSV-LCMVG. **g**. Tumor volumes are shown as mean values with SEM. Survival curves of C57BL/6J mice from the experiment described in (**f**) are shown. Tumor response data derived from mice (n = 5) are shown. *p < 0.05; **p < 0.01; ***p < 0.001; ****p < 0.0001, based on two-way ANOVA with post hoc Holm-Sidak test; survival analysis was conducted using log rank test. **h.** Tumor volumes for each mouse in each treatment group are shown for mice described in (**g**).


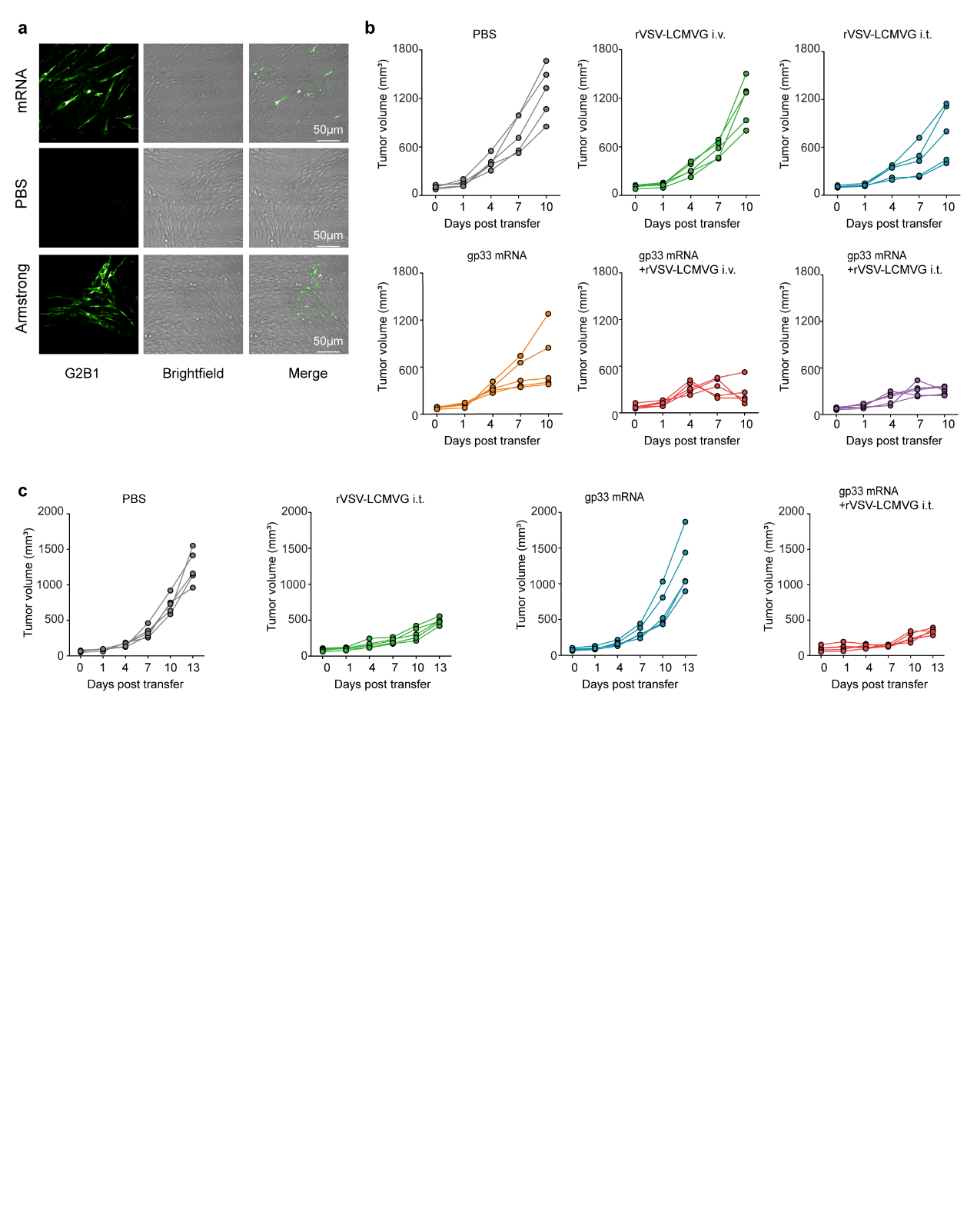


**Figure. S8. Combining GP33 mRNA Vaccine With Oncolytic Viruses In Tumor Treatment.**

**a**. Immunofluorescence demonstrating that GP33 mRNA could express gp33 protein at the cellular level in vitro. **b.** Tumor volumes for each mouse in each treatment group are shown for mice described in (Figure 6**f**)**. c**. Tumor volumes for each mouse in each treatment group are shown for mice described in (Figure 6**h**) .
